# The Stabilization of High‐Nitrogen Systems: 10 Catenated Nitrogen Chain

**DOI:** 10.1002/chem.202503256

**Published:** 2026-05-06

**Authors:** Nicholas F. Scherschel, Thomas R. Talbot, Matthias Zeller, Davin G. Piercey

**Affiliations:** ^1^ Purdue Energetics Research Center West Lafayette Indiana USA; ^2^ School of Materials Engineering Purdue University West Lafayette Indiana USA; ^3^ Department of Chemistry Purdue University West Lafayette Indiana USA; ^4^ School of Mechanical Engineering Purdue University West Lafayette Indiana USA

**Keywords:** azo compounds, catenated nitrogen chain, high‐nitrogen heterocycles, tetrazoles

## Abstract

High‐nitrogen materials have attracted intense interest in recent decades due to their unique chemical, physical, and biological properties. Stabilizing high‐nitrogen materials is often challenging as they become more unstable with increasing nitrogen catenation and nitrogen mass percentage increase.  Although approaches such as introducing alternating positive/negative charges and restricting electrocyclic N_2_ loss have been reported for stabilizing high‐nitrogen systems, these structural motifs have not previously been applied to highly catenated nitrogen chains. Accordingly, we sought to design a new highly catenated nitrogen chain with the aforementioned considerations, which began with the synthesis of 1,5‐diamino‐3‐methyl‐tetrazolium chloride. When 1,5‐diamino‐3‐methyl‐tetrazolium chloride is reacted with white fuming nitric acid, a novel 10‐catenated nitrogen chain in 1,1’‐azobis‐3,3’‐dimethyl‐5,5’‐diamino‐tetrazol‐3,3’‐ium dinitrate is afforded. Physical and chemical properties of this exotic material are described herein, and implications for high‐nitrogen material design are discussed.

## Graphical abstract

Improving the understanding of high‐nitrogen materials.

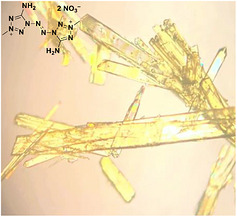

## 1 Introduction

Highly catenated nitrogen systems, especially azobis(tetrazoles) have been a topic of intense study the last two decades [1, 2] as they present an interesting quandary for inorganic chemists—trading higher degrees of catenation for lower thermal and mechanical stability. In the 1990s, Shreeve, Krumm et al. synthesized saturated perfluorinated‐tetrazanes from the corresponding *N*‐chlorohydrazines by photolysis [3, 4]. These acyclic tetrazanes contain four contiguous nitrogen atoms and are unusually hydrolytically as well as thermally stable. The longest neutral catenated nitrogen material, 1,1’‐azobis(tetrazole), was documented in 2011 by Klapötke and Piercey, and has gathered exceptional interest since its publication, with nearly 300 citations at the time of writing [5]. The longest nitrogen chain, 1,1’‐(triaz‐1‐ene‐1,3‐diyl)bis(1H‐tetrazol‐5‐amine), was synthesized in 2013 by Cheng and coworkers [6], which features a monocationic 11‐nitrogen chain. For additional discussion on highly catenated nitrogen systems, see T. M. Klapötke's *Chemistry of High‐Energy Materials*, seventh edition [7].

High‐nitrogen compounds are useful in a wide range of fields, and tetrazoles are one of the most widely used high‐nitrogen content heterocyclic building blocks due to several unique characteristics. First, tetrazoles generally exhibit low toxicity and are even used in medicinal chemistry as a bioisostere for carboxylic acids, as tetrazoles are not metabolized in the liver while carboxylic acids are [8]. This differentiates tetrazoles from the other “lower” azoles, such as 1,2,4‐triazoles [9, 10, 11], 1,2,3‐triazoles [12, 13, 14], pyrazoles [15, 16, 17], and imidazoles [10, 18, 19] which are also bioisosteres, though the lower nitrogen azoles tend to have more toxicity concerns than tetrazoles [20, 21]. Second, growing interest in sustainable nitrogen fixation and greener synthetic routes has enabled environmentally friendly preparation of the tetrazole skeleton [22], expanding the potential of tetrazoles and high‐nitrogen compounds for energy storage applications [23]. As tetrazoles inherently possess high enthalpies of formation due to the large nitrogen content in their backbone, they can be considered as a form of energy storage. Release of the potential energy is achieved by decomposition of the parent tetrazole, and in so doing, evolving environmentally benign N_2_ gas. Some high‐nitrogen compounds are also capable of decomposing very expediently and are potent energetic materials [24, 25, 26].

**FIGURE 1:**
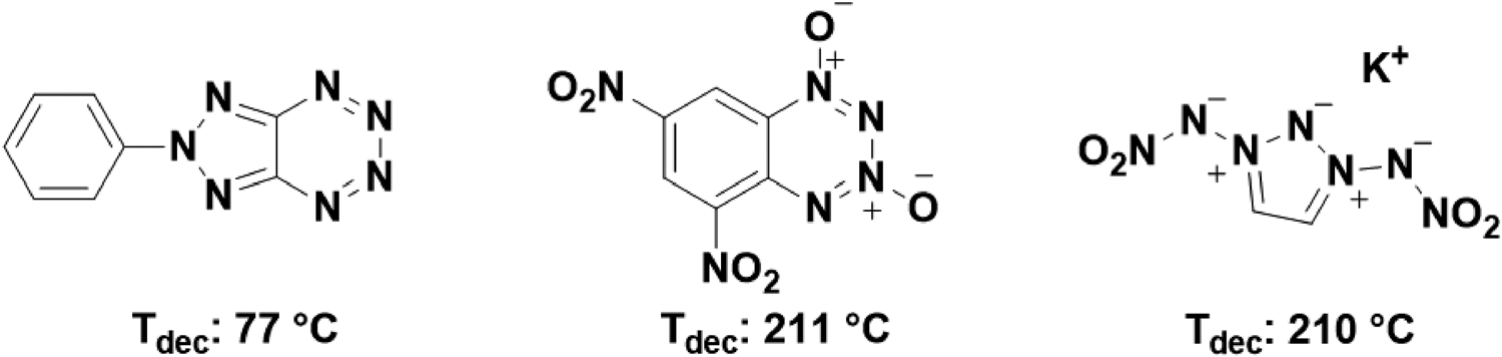
Chemical structures of 6‐phenyl [1,2,3]triazolo[4,5‐e]‐1,2,3,4‐tetrazine (left) [28], 5,7‐dinitro‐benzotetrazine 1,3‐dioxide (middle) [29], and potassium 1, 3‐bis(nitroimido)‐1, 2, 3‐triazolate (right) [31] with their thermal decomposition temperatures noted.

Although high‐nitrogen systems have a diverse range of applications, their thermal (and often mechanical) stability typically decreases with increasing nitrogen content in a molecule [5, 27]. That being said, there are means to improve the thermal stability of high‐nitrogen systems. First, if alternating positive and negative charges (APNC) are introduced into the backbone, stability is usually improved. An excellent example of APNC improving the thermal stability of a material is the comparison between a 1,2,3,4‐tetrazine and a 1,2,3,4‐tetrazine‐1,3‐di‐*N*‐oxide. Comparing 6‐phenyl[1,2,3]triazolo[4,5‐e]‐1,2,3,4‐tetrazine to its 1,2,3,4‐tetrazine dioxide counterpart in 5,7‐dinitro‐benzotetrazine 1,3‐dioxide (Figure 1), reveals decomposition temperatures of 77°C [28] and 211°C [29], respectively. Indeed, the difference of over 100°C between the 1,2,3,4‐tetrazine and its 1,2,3,4‐tetrazine‐1,3‐di‐*N*‐oxide is impressive. Important factors thought to yield improved stability are the tetrazine dioxide's lack of ring‐chain tautomerism, introduction of alternating charges in the tetrazine dioxide, and inhibition of electrocyclic extrusion of N_2_ gas by the 1,3 substitution pattern in 1,2,3,4‐tetrazine‐1,3‐di‐*N*‐oxides [29, 30]. Another example of the thermal stability conferred by the APNC principle is found in potassium 1, 3‐bis(nitroimido)‐1, 2, 3‐triazolate (Figure 1, right), which has a remarkable thermal stability of 210°C despite its seven‐nitrogen chain [31].

**FIGURE 2:**
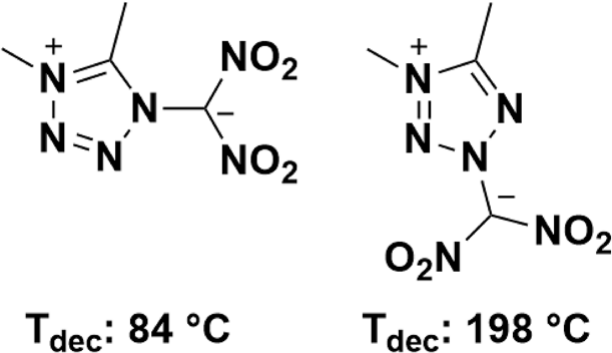
Chemical structures of (4,5‐dimethyl‐1H‐tetrazol‐4‐ium‐1‐yl)dinitromethanide (left) and (4,5‐dimethyl‐2H‐tetrazol‐4‐ium‐2‐yl)dinitromethanide (right) with thermal decompositions noted [32].

Another approach to stabilizing high‐nitrogen systems, with emphasis on tetrazoles using 1,2,3,4‐tetrazines as an analogy, is by substituting their backbones to prevent the electrocyclic extrusion of N_2_ gas from the parent heterocycle—analogous to how 1,2,3,4‐tetrazine‐1,3‐di‐*N*‐oxides are stabilized as discussed above. An example stressing the importance of a tetrazole's substitution pattern for its thermal stability is the comparison of (4,5‐dimethyl‐1H‐tetrazol‐4‐ium‐1‐yl)dinitromethanide (Figure 2, left) with (4,5‐dimethyl‐2H‐tetrazol‐4‐ium‐2‐yl)dinitromethanide (Figure 2, right). These tetrazoles are constitutional isomers of each other; however, the 1,4,5‐substituted tetrazole possesses a thermal decomposition of just 84°C, while its 2,4,5‐substituted counterpart has a thermal decomposition temperature of 198°C—a notable improvement relative to its 1,4,5‐substituted counterpart! [32]

A previously prepared tetrazole, 1,1’‐azobis(tetrazole), lacks all the aforementioned stabilizing motifs and has a thermal decomposition temperature of 80°C [5]. To see if a more stable N_10_ system may be synthesized, we sought to prepare a 1,3,5‐trisubstituted tetrazole with an N─NH_2_ group on the *N*_1_ position, as these tetrazoles form N_10_ chains when azo‐coupled on the *N*‐amine. Since 5‐amino‐2‐methyl‐tetrazole is a commercially available precursor, we performed the amination of this material to obtain 1,5‐diamino‐3‐methyl tetrazolium chloride (**1**), a previously unidentified tetrazole, although an isomer of the previously described 2,5‐diamino‐4‐methyl‐tetrazolium and related to 2,4,5‐triamino‐tetrazolium [33].

## 2 Results and Discussion

### 2.1 Experimental Details

*Caution!* Compounds **2–6** in this work are sensitive energetic materials. When working with the dried material, Kevlar body armor, gloves, and a neck guard are worn in addition to a face shield. Dry materials are stored in plastic beakers and manipulated with plastic spatulas only when necessary.

**SCHEME 1:**
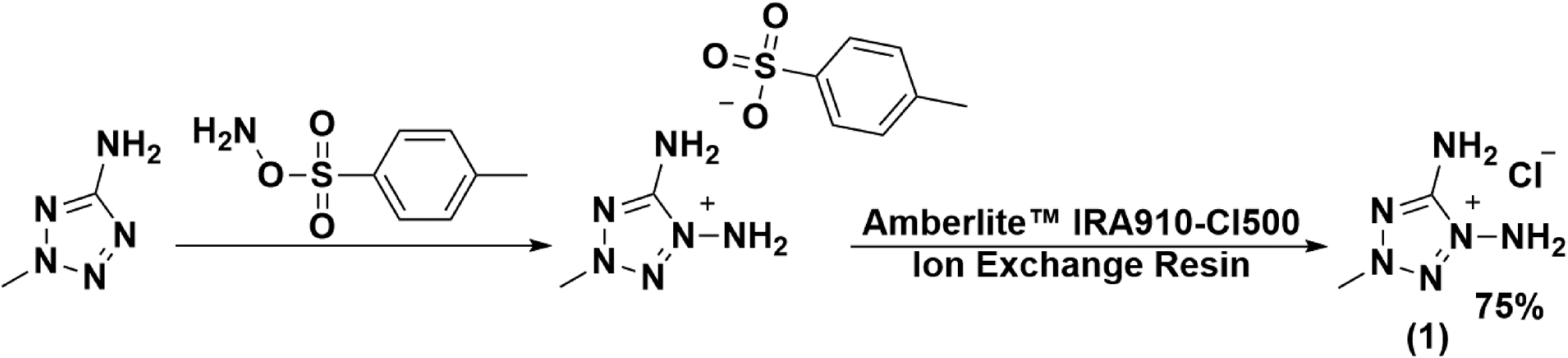
Synthesis of **1** from 5‐amino‐2‐methyl‐tetrazole.

To prepare **1**, the commercially available 5‐amino‐2‐methyl‐tetrazole was first aminated with *O*‐tosylhydroxylamine in a DCM/MeCN mixture [24]. Next, the crude tosylate salt of tetrazole **1** was filtered from the solution and dried. Purification of the tosylate salt of **1** was achieved by dissolving the tosylate salt of **1** in a minimum amount of hot ethanol, followed by decanting the solution into 10 volume equivalents of pentane, from which the tosylate salt of **1** precipitates as a fine white powder. This powder is filtered, dried, then redissolved in water and treated with Amberlite IRA910‐Cl500 to exchange the tosylate anion for a chloride anion. After ion exchange is complete, the water is removed by blowing a stream of air over the solution, whereupon **1** crystalizes in large light‐yellow crystals suitable for single crystal x‐ray diffraction. A summary of the synthesis is shown in Scheme 1, below. For exact experimental details, see the Supporting Information.

**SCHEME 2:**
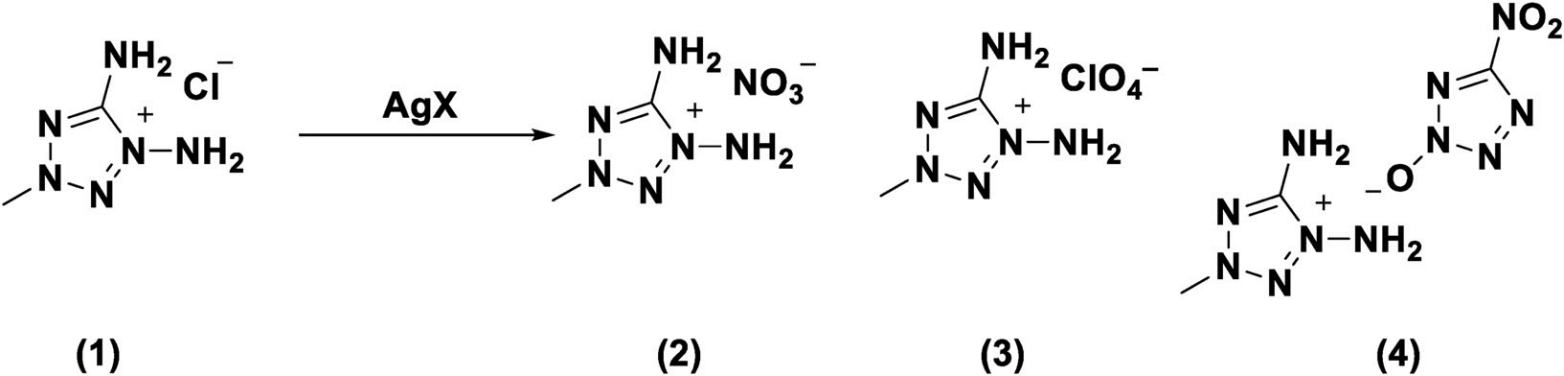
Synthesis of salts **2–4** by silver salt metathesis, where X is NO_3–_ for **2**, ClO_4–_ for **3**, and 5‐nitrotetrazolate‐2*N*‐oxide for **4**.

After **1** was isolated, salts were made by silver salt metathesis with silver nitrate, silver perchlorate, or silver nitrotetrazolate to obtain 1,5‐diamino‐3‐methyl tetrazolium nitrate (**2**), 1,5‐diamino‐3‐methyl tetrazolium perchlorate (**3**), and 1,5‐diamino‐3‐methyl tetrazolium (5‐nitrotetrazolate‐2*N*‐oxide) (**4**), respectively (Scheme 2). Generally, about 100 mg of **1** was dissolved in 10 mL of water in a plastic beaker, whereupon one equivalent of the relevant silver salt was added. The reaction was stirred for at least an hour in darkness, whereupon the precipitated silver chloride was removed by filtration. Lastly, the filtrate is placed in a plastic beaker and dried under an air stream to afford a quantitative yield (99%+) of the respective salt.

**FIGURE 3:**
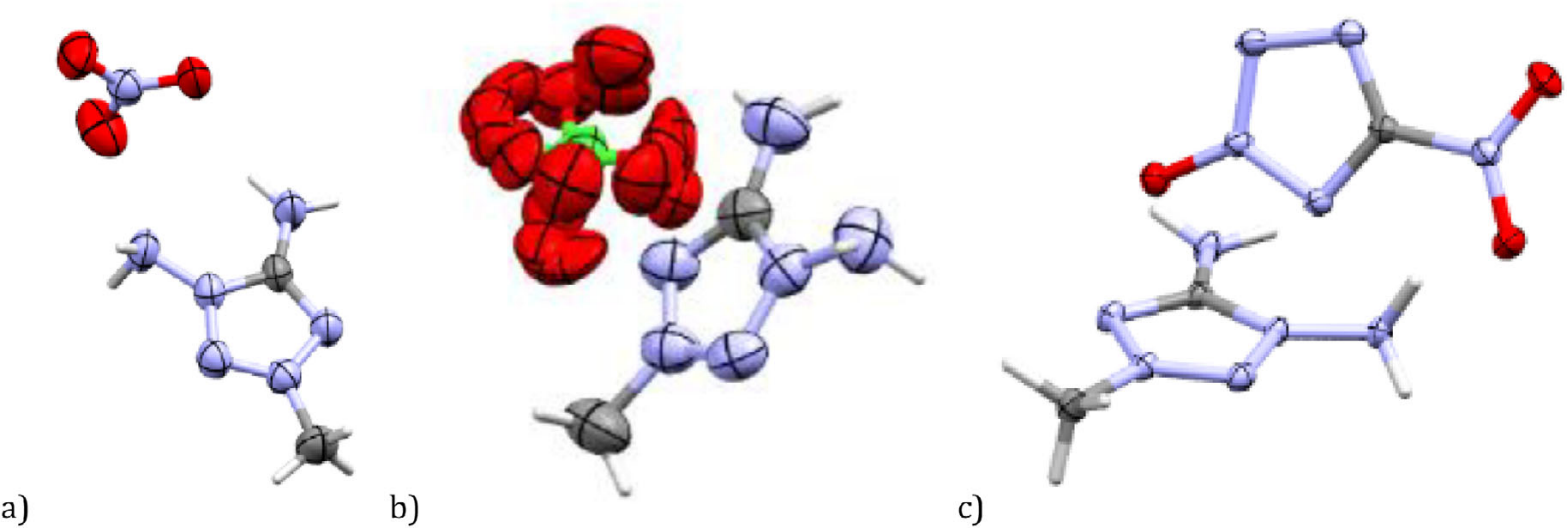
Molecular structures of **2** (a), **3** (b), and **4** (c) as they appear in their crystal structures. Non‐hydrogen atomic displacement ellipsoids are shown at 50% probability.

Crystals suitable for single crystal x‐ray diffraction of **2** and **3** were obtained by MeOH/Et_2_O diffusions, while suitable crystals of **4** were obtained by slow evaporation from water. Single crystal x‐ray diffraction afforded structures of **2**–**4**, which may be seen in Figure 3, below. No disorder was observed in **2** and **4**; however, **3** demonstrated significant disorder around the perchlorate anion, which is shown in Figure 3b. Space groups were found to be *Cc*, *Pbca*, and *P*_21/*n*_ for **2–4**, respectively.

When **1** was reacted with white fuming nitric acid, it was found to afford several products, depending on the workup. The most interesting product is 1,1’‐azobis‐3,3’‐dimethyl‐5,5’‐diaminotetrazol‐3,3’‐ium dinitrate (**5**), a dicationic material possessing a 10‐nitrogen chain. To the best of our knowledge, this is the first 10‐nitrogen chain which is dicationic. **5** was synthesized by chilling 1 mL of white fuming nitric acid to 0°C using an ice bath, then adding 100 mg of **1** slowly to the fuming nitric acid. The solution was allowed to stir for 30 min, whereupon it was poured into 10 mL of chilled water in a plastic beaker, which was subsequently placed under an air stream. As the solution was drying, gas evolution was observed in the solution as small bubbles, which was posited to be the decomposition of **5**. Once the solution was dried completely, **5** crystallized as orange rods in 30% yield. Fortuitously, these rods were suitable for single crystal x‐ray diffraction. Attempts to exchange the nitrate anion in **5** for other anions using ion exchange resin were unsuccessful, leading only to decomposition products being obtained (ammonium perchlorate in the case of perchlorate ion exchange). Additionally, when **5** was dissolved in D_2_O for NMR, bubbles were observed to form again, which points to the instability of **5** in solution.

**SCHEME 3:**
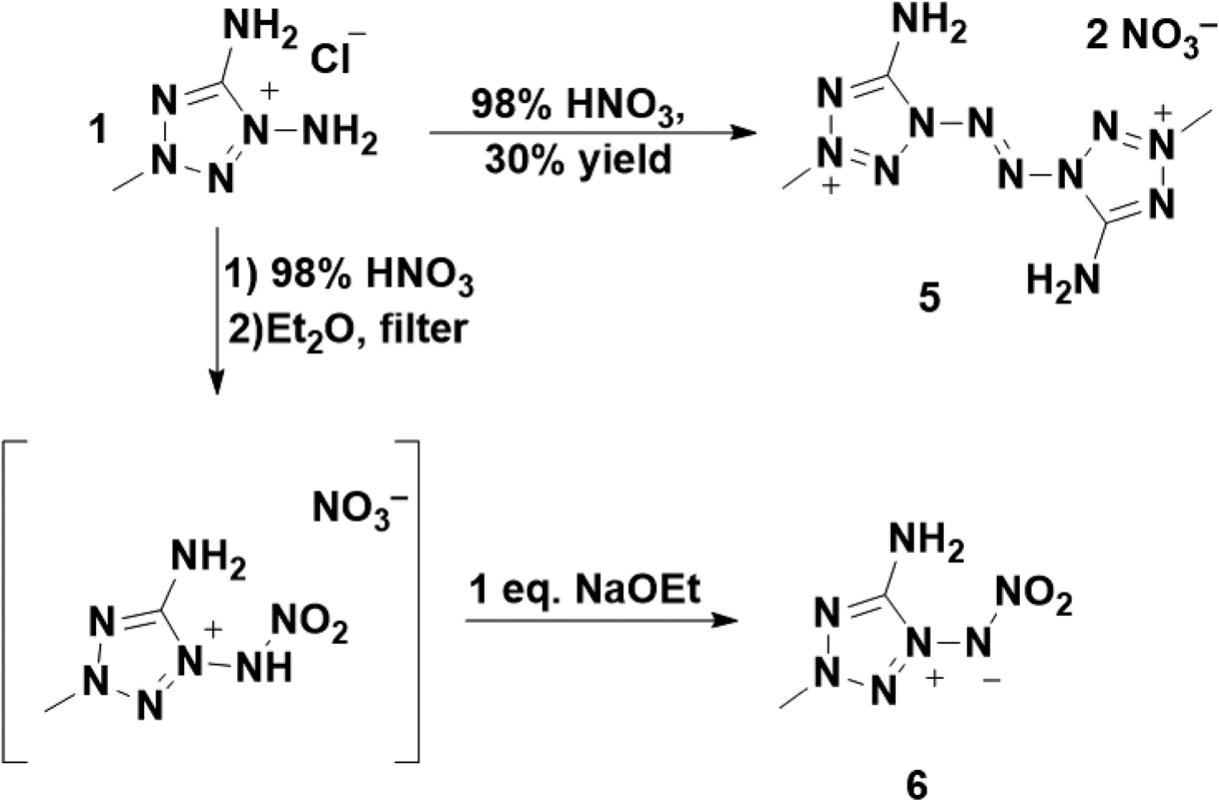
Synthesis of **5** by nitration of **1** in white fuming nitric acid with aqueous workup. **6** was acquired by an Et_2_O workup.

An additional product, 5‐amino‐2‐methyl‐4‐nitriminotetrazole (**6**), was isolated by pouring the solution of (**1**) in 1 mL of fuming nitric acid into 20 mL of Et_2_O, whereupon a bright yellow precipitate formed. The precipitate was filtered and dried, then suspended in 10 mL EtOH, and one equivalent of ethanolic sodium ethoxide was added. Immediately upon addition of the basic solution, the suspension changed color from yellow to orange. After the addition of the ethoxide, the solution was allowed to stir for an hour, then the inorganic precipitates were removed by filtration, and the solution was dried under an air stream to yield **6** as an orange powder. Suitable crystals for single crystal x‐ray diffraction of **6** were obtained by slow evaporation from ethanol, acetone, as well as an ethanol/methyl‐tert‐butyl ether diffusion. A summary of the reaction is shown below in Scheme 3.

**FIGURE 4:**
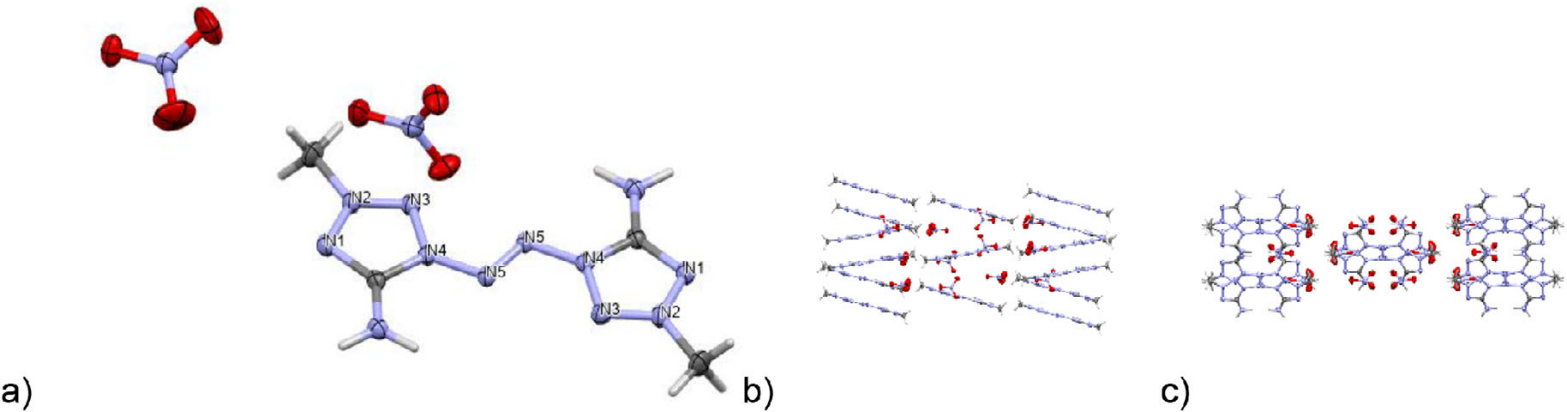
(a) Molecular structure of **5** as it appears in its crystal structure with contiguous nitrogen atoms numbered. Non‐hydrogen displacement ellipsoids are shown at 50% probability. (b) Side view of **5** as it packs in its crystal structure. (c) Top‐down view of **5** as it appears in its crystal structure.

Single crystal x‐ray diffraction of **5** elucidated the structure and packing, which is shown in Figure 4. Upon examination of the bond lengths in the structure of **5**, it is noted that the azo *N*─*N* double bond length is 1.247 ± 0.002 Å, which is significantly longer than in its neutral relative, 1,1’‐azobis(tetrazole) (**7**), which has an azo *N*─*N* double bond length of 1.178 Å [5]. Notably, the *N*─*N* single bond between *N*_4_ and *N*_5_ (Figure 4, a) is remarkably shorter for compound **5** relative to compound **7**, with bond lengths of 1.367 ± 0.002 and 1.459 Å, respectively. All other *N─N* bond lengths are comparable to those of other 1‐substituted tetrazole systems [34]. A summary of the *N*─*N* bond lengths for **5** is given in Table 1, below.

**TABLE 1:** Summary of *N*─*N* bond lengths of **5** in Å.

*N─N* Bond	*N─N* Bond Length (± 0.002Å)
*N*_1_ *─N*_2_	1.339
*N*_2_ *─N* **_3_**	1.284
*N*_3_ *─*N_4_	1.352
N_4_ *─N*_5_	1.366
*N*_5_ *─*N_5’_	1.247

**FIGURE 5:**
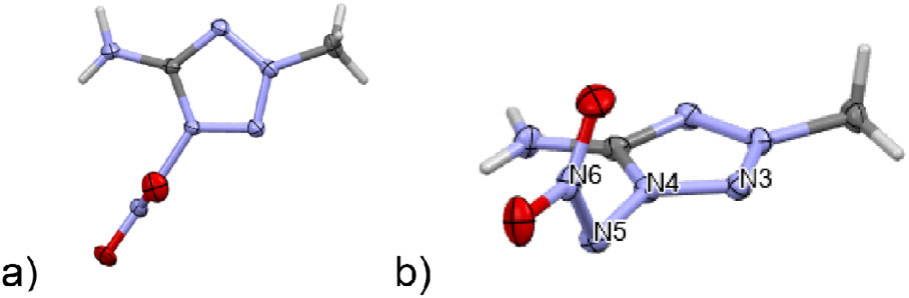
(a) and (b) are the molecular structure of **6** as it appears in its crystal structure, with non‐hydrogen displacement ellipsoids shown at 50% probability. (a) top‐down view of **6**, (b) side view of **6** with *N*_6_ *─N*_5_ *─N*_4_ *─N*_3_ numbered for clarity on the torsionl angle.

**FIGURE 6:**
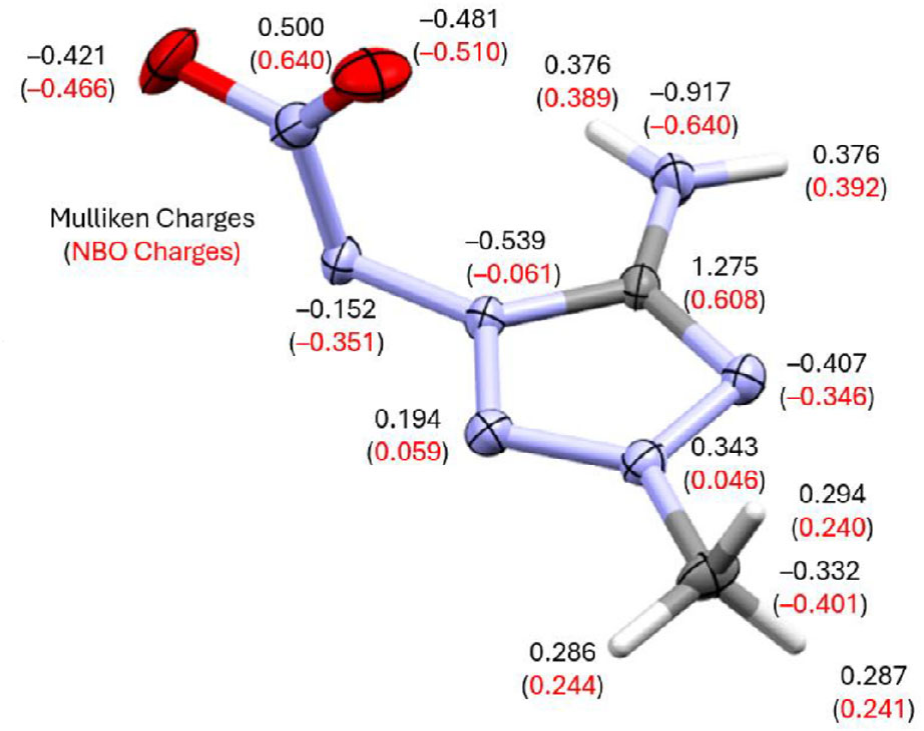
Calculated Mulliken and NBO charges (red) for compound **6**.

The crystal structure of **6** was elucidated with single crystal x‐ray diffraction, and its structure can be seen in Figure 5. Remarkably, the torsion angle for the nitrimine relative to the tetrazole is nearly orthogonal, with *N*_6_─*N*_5_─*N*_4_─*N*_3_ possessing a dihedral angle of 92.3° (see Figure 5b for numbering of relevant nitrogen atoms). The density of **6** was found to be 1.68 g/cm^3^ by a single crystal X‐ray diffraction measurement at 22°C. Further elucidation of the charge distribution on compound **6** is shown in Figure 6, where calculated Mulliken and natural bond orders (NBOs) are displayed. Calculations are evaluated at the B3LYP/cc‐PVTZ level of theory using an optimized gas‐phase structure in Gaussian16 software [35], consistent with a previous report by Klapötke [31]. An unusual feature found in the Mulliken and NBO charges found in compound **6** is two groups of like‐charges adjacent to each other in the *N*_5_─*N*_4_ pair, which have calculated Mulliken charges of −0.152 and −0.539, while NBO are found to be −0.351 and −0.061, respectively. Another unfavorable interaction is evident within the ring between the amine bonded to the methyl group, *N*_2_, and *N*_3_. Here, the Mulliken values are 0.343 and 0.194 for *N*_2_ and *N*_3_, respectively, while the NBO charges are 0.046 and 0.059, respectively, again. In a “true” alternating charge system, there would be no adjacent atoms with a “like” charge in the ring, so these calculations aid in describing some destabilizing interactions.

### 2.2 Physical and Energetic Properties

Compounds **2–6** are all highly sensitive and explosive materials, and care should be taken in their handling. Of these, **5** and **6** are the most sensitive with impact and friction sensitivities of < 1 J and < 5 N, respectively. Compound **2** possesses a similar impact sensitivity of < 1 N; however, the compound is not as hypersensitive to friction stimuli, with a value of 23 N found. Compound **3** was found to have an impact sensitivity of 1 J and a friction sensitivity of 5 N, and compound **4** has an impact sensitivity of 4 J while its friction sensitivity is < 5 N. All compounds are significantly more sensitive than TNT, which has an impact sensitivity of 15 J and a friction sensitivity of 360 N [36]. Though compound **5**’s sensitivity is below the limit of detection for our instruments, we did not experience accidental detonation of this material. In contrast, 1,1′‐azobis(tetrazole) (**7**) did undergo multiple unexpected detonations during its synthesis [5], which could indicate compound **5** is slightly more insensitive, albeit it certainly remains a material highly sensitive to mechanical stimuli. A summary of all energetic properties for compounds **2** **–6** is available in Table 1.

Thermal decomposition temperatures of all materials were determined by thermogravimetric analysis (TGA). Of the diamino methyl tetrazolium salts, compounds **2** and **4** are comparable at 149°C and 147°C, respectively. Compound **3** has a decomposition temperature of 166°C, the highest of the energetic diamino methyl tetrazolium salts in this work. Some comparison to known constitutional isomers is possible with compounds **2** and **3**, as they are described elsewhere [33]. While **2** has a thermal decomposition of 149°C, its isomer, 2,5‐diamino‐4‐methyl tetrazolium nitrate, decomposes at a higher temperature of 203°C. On the other hand, **3** decomposes at 166°C, while its isomer in 2,5‐diamino‐4‐methyl tetrazolium perchlorate decomposes slightly lower at 155°C. The azo‐coupled counterpart of **2**, compound **5**, has a markedly lower thermal decomposition at 93°C, likely due to the inherent instability of such highly catenated nitrogen systems. Nevertheless, it is worthwhile noting that compound **5**’s 93°C thermal decomposition temperature is slightly higher than that of compound **7**’s at 80°C [5]. Lastly, the thermal decomposition of compound **6** was found to be 170°C, which is slightly higher than its isomer, 5‐amino‐4‐methyl‐1‐nitriminotetrazole, which decomposes at 150°C [37].

Single crystal x‐ray diffraction studies of compounds **2**–**6** at room temperature determined the densities of each material. Of the diamino methyl tetrazolium salts, compounds **3** and **4** were found to have similar densities at 1.67 and 1.68 g/cm^3^, respectively, while **2** has the lowest density at 1.54 g/cm^3^. Azo‐coupling **1** to afford **5** increases the density to 1.69 g/cm^3–^ the highest in this series, while **6** has a comparable density to **3** and **4** at 1.68 g/cm^3^. Complete crystallographic details for **1**–**6** are deposited within the CCDC database.

Analysis of each compound's energetic properties was conducted with a hybrid computational–experimental approach, where the experimentally found density from single crystal x‐ray diffraction was used in conjunction with computationally identified enthalpies of formation. Enthalpies of formation were calculated with the CBS‐4M methodology utilizing the Gaussian 16 suite according to previously reported methodology [33, 38, 39]. Once the calculated enthalpy of formation was identified, the value was used in conjunction with the single crystal X‐ray diffraction density in the Explo5 V6.05.02 software to determine the theoretical detonation velocity and detonation pressure [40].

Calculated enthalpies of formation were found to be 250.4, 193.8, 663.5, 628.6, and 433 kJ/mol for compounds **2**–**6**, respectively. Of note, **5** has an enthalpy of formation of 402 kJ/mol, lower than its neutral N_10_ counterpart **7**, likely attributable to **5**’s ionic interactions lowering the enthalpy of formation, as well as the introduction of methyl groups in **5**.

Results of the Explo5 calculations indicate compound **2** has the lowest energetic performance in the series, with a calculated detonation velocity of 8205 m/s and a detonation pressure of 24.7 GPa. Compound **3** has a slightly higher detonation pressure at 26.6 GPa, while its detonation velocity is slightly lower at 8076 m/s. Compounds **4**, **5**, and **6** are remarkably similar, with detonation pressures of 28.7 GPa for **4**, **5,** the highest at 29.4 GPa, and **6** at 28.8 GPa. Detonation velocities for **4–6** are 8686, 8645, and 8683 m/s, respectively. Though **4–6**’s detonation performances exceed that of TNT, they fall short of **7**. Calculated detonation parameters are provided for completeness, and compounds **2–6** are not intended for use as practical explosive materials, rather as exemplification of the strategies and results from nitrogen chain stabilization (Table 2).

**TABLE 2:** Summary of energetic performances of compounds **2**–**6**.

Material
	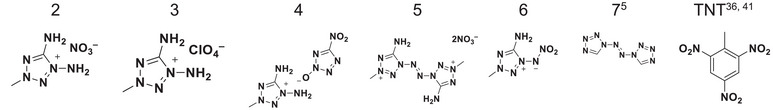						
IS (J)^a^	< 1	1	4	< 1	< 1	< 1	15
FS (N)^b^	23	5	< 5	< 5	< 5	< 5	> 360
*N* (%)	55.3	39.2	62.8	56.0	51.9	84.3	18.5
*Ω*_CO_ (%)	−40.7	−22.4	−42.4	−32.0	−45.2	N/A	−71.0
*T*_dec._ (°C)^c^	149	166	147	93	170	80	295
Density (g/cm^3^)^d^	1.54	1.67	1.68	1.69	1.68	1.77	1.65
Δ_f_ *H* (kJ/mol)^e^	250.4	193.8	663.5	628.3	433	1030	−63.2
*P*_C‐J_ (GPa)^f^	24.7	26.6	28.7	29.4	28.8	36.1	19.5
*D* (m/s)^f^	8205	8076	8686	8645	8683	9185	6881
Heat of Detonation (K)	3405	3766	3576	3640	3570	4209	3218

^a^ Impact sensitivities identified by STANAG 4489 [42] with modifications to an OZM drop hammer by the BAM method, according to instruction [43].

^b^ Friction sensitivities are conducted with STANAG 4487 [44] with modification to instruction [45] on a BAM friction tester.

^c^ Thermal decomposition taken from the onset of mass loss on the TGA curve.

^d^ Densities of **2**–**6** were determined from room temperature single crystal x‐ray crystallography measurements.

^e^ Enthalpy of formation is calculated with the CBS‐4M methodology and is consistent with previous reports [35, 38, 39].

^f^ Detonation pressures, velocities, and detonation temperatures were calculated with the Explo5 V6.05.02 [40] software suite using the experimentally determined room temperature single crystal x‐ray density in conjunction with the CBS‐4M calculated enthalpy of formation.

## 3 Conclusion

Designing stable high‐nitrogen content systems is a challenging endeavor, though incorporating certain design motifs, such as alternating charges and hindrance of electrocyclic N_2_ loss by substitution pattern, are likely critical to continued progress on the matter. With these concepts in mind, we designed and synthesized 1,5‐diamino‐3‐methyl‐tetrazol‐3‐ium chloride (**1**) and azo coupled it to afford the exotic 1,1’‐azobis‐3,3’‐dimethyl‐5,5’‐diaminotetrazol‐3,3’‐ium dinitrate (**5**), which has improved thermal stability over its neutral counterpart 1,1’‐azobis(tetrazole) (**7**), illustrating the possibility of tailoring high‐nitrogen systems for thermal stability.

## Funding

Financial support for this work was provided by the Office of Naval Research under grant N00014–19–1–2089. Our lab is also supported by Purdue University and the Army Research Office (ARO).

## Conflicts of Interest

The authors declare no conflicts of interest.

## Supporting information



CCDC 2483220, 2483215, 2483218, 2483216, 2483217, 2483219 contain the supplementary crystallographic data for this paper. These data can be obtained free of charge from The Cambridge Crystallographic Data Centre via www.ccdc.cam.ac.uk/structures. **Supporting File**: chem71047‐sup‐0001‐SuppMat.docx.

## Data Availability

The data that support the findings of this study are available in the Supporting Information of this article.
