# Supplementary material for: The Stabilization of High‐Nitrogen Systems: 10 Catenated Nitrogen Chain
Source: Chemistry. 2026 May 6;32(31):e03256. doi: 10.1002/chem.202503256 (PMC13485250; doi:10.1002/chem.202503256)
Supplement: Supplementary file 1 [file CHEM-32-e03256-s001.docx]

Supplemental Information

The Stabilization of High-Nitrogen Systems: 10 Catenated Nitrogen Chain

Nicholas F. Scherschel,^1,2^ Thomas R. Talbot IV ^2^ Matthias Zeller,^3^ Davin G. Piercey^1,2,4*^

^1^School of Materials Engineering West Lafayette, IN 47907, USA

^2^Purdue Energetics Research Center, Purdue University, West Lafayette, IN 47906, USA

^3^Department of Chemistry, Purdue University, West Lafayette, IN 47907, USA

^4^School of Mechanical Engineering, Purdue University, West Lafayette, IN 47907.

Table of Contents

1. General Procedures, Instruments, and Methods	2
2. Synthesis of 4,5-diamino-2-methyl-2H-tetrazol-4-ium chloride (**1**)	2
3. Synthesis of 4,5-diamino-2-methyl-2H-tetrazol-4-ium nitrate (**2**)	2, 3
4. Synthesis of 4,5-diamino-2-methyl-2H-tetrazol-4-ium perchlorate (**3**)	3
5. Synthesis of 4,5-diamino-2-methyl-2H-tetrazol-4-ium 5-nitrotetrazolate-2-*N*-oxide (**4**)	3
6. Synthesis of 1,1’-azobis-3,3’-dimethyl-5,5’-diaminotetrazol-3,3’-ium dinitrate (**5**)	3
7. Synthesis of 5-amino-2-methyl-4-nitriminotetrazole (**6**)	3
8. References	4
9. Crystallographic Details	5–28

General Procedures, Instruments, and Methods.

Ethyl-O-p-tolyslsulfonyl-acetohydroximate^46^ and silver 5-nitrotetrazolate-2-*N*-oxide^26^ were prepared according to literature procedure. Silver perchlorate, silver nitrate, 5-amino-2-methyl tetrazole, sodium ethoxide, and white fuming nitric acid were used as received. A Bruker AV-III-500-HD (5 mm BBFO Cryoprobe Prodigy) Avance DRX NMR spectrometer reported ^1^H NMR and ^13^C NMR for all materials. IR was recorded on a PerkinElmer Spectrum Two FT-IR spectrometer with transmittance noted as strong (s), medium (m), and weak (w). Elemental analysis for compounds **1**-**6** is not reported due to the explosive decomposition of all compounds at elevated temperatures possibly damaging the sensitive elemental analysis instrumentation, which we have previously experienced with similar compounds.

Crystallographic data for all materials are collected from a shock-cooled single crystal at 150(2) K on a Bruker AXS D8 Quest three circle diffractometer with a fine focus sealed tube X-ray source using a Triumph curved graphite crystal as monochromator and a PhotonII charge-integrating pixel array (CPAD) detector. The diffractometer used Mo*K_α_* radiation (λ = 0.71073 Å). All data were integrated with SAINT V8.40B and a multi-scan absorption correction using SADABS 2016/2 was applied.^47, 48^ The structure was solved by dual methods with SHELXT and refined by full-matrix least-squares methods against *F*^2^ using SHELXL-2019/2.^49, 50^ All non-hydrogen atoms were refined with anisotropic displacement parameters. All hydrogen atoms were refined with isotropic displacement parameters. Some of their coordinates were refined freely and some on calculated positions using a riding model with their *U*_iso_ values constrained to 1.5 times the *U*_eq_ of their pivot atoms for terminal sp^3^ carbon atoms and 1.2 times for all other carbon atoms. Crystallographic data for the structures reported in this paper have been deposited with the Cambridge Crystallographic Data Centre.^51^ CCDC 2483220, 2483215, 2483218, 2483216, 2483217, and 2483219 contain the supplementary crystallographic data for this paper. These data can be obtained free of charge from The Cambridge Crystallographic Data Centre via www.ccdc.cam.ac.uk/‌structures. This crystallographic tables included herein and the CIF files were generated using FinalCif.^52^ CBS-4M enthalpy of sublimation estimated to be 180 °C for all materials.

S1. Synthesis of 4,5-diamino-2-methyl-2H-tetrazol-4-ium chloride (**1**).

In a round bottom flask, 1.1 equivalents (1.43g) of ethyl-O-p-tolylsulfonyl-acetohydroximate is added to ~25 mL of 60% perchloric acid at room temperature. The solution is stirred for two hours, then poured onto ~150 mL of ice water. The white aminating agent is extracted with 3x30 mL dichloromethane, whereupon the organic extracts were combined and dried over magnesium sulfate. Next, the DCM extracts were decanted into a round bottom flask, and 1 eq. of 5-amino-2-methyl-tetrazole (0.500g) dissolved in acetonitrile is added to the aminating solution dropwise. After about an hour, a white precipitate forms. The reaction is allowed to stir for at least 16 hours, whereupon the precipitate is separated by filtration. Next, the precipitate is added to a beaker where about 10 mL of ethanol were added. The crude white precipitate is then recrystallized by heating the solution until boiling and adding sufficient ethanol to completely dissolve all solids. Once dissolved, the ethanol is poured into pentane where the tosylate salt of 4,5-diamino-2-methyl-2H-tetrazol-4-ium precipitates as a fine white powder. The precipitate is filtered, dried, then dissolved in water and added to ion exchange beads loaded with Cl^–^. Ion exchange beads are replaced three times, and the exchange is deemed complete when no tosylate anion is observed on CI-MS. After no evidence of OTs^–^ is found, the aqueous solution containing 1,5-diamino-3-methyltetrazol-3-ium chloride (**1**) is dried under an airline in a plastic beaker, where **1** crystallizes as light-yellow needle-like crystals which are suitable for single crystal X-ray diffraction. Yield: 759 mg (75.0%). ^1^H NMR (D_2_O) δ 4.3 ppm (s) ^13^C (D_2_O) NMR δ 157.3, δ 43.1 ppm. HRMS calculated (M^+^) 115.0726704, found 115.07235. ^15^N NMR showed decomposition of compound **1** over the required timescale for ^15^N NMR collection of unenriched material (~14 hours).

S2. Synthesis of 4,5-diamino-2-methyl-2H-tetrazol-4-ium nitrate (**2**).

In plastic beaker, 100 mg of **1** is dissolved in ~10 mL of water. Next, one equivalent of AgNO_3_ (0.148 g) is dissolved in a minimum amount of water and added dropwise to the solution of **1**. Upon addition, a white precipitate forms and the solution is stirred for one hour, then the precipitate is separated by filtration, and the filtrate placed in a plastic beaker and dried under an airline. Crystals of **2** suitable for single crystal X-ray diffraction are grown by a diffusion of Et_2_O into a solution in MeOH. Yield: 116 mg (99%). ^1^H NMR (D_2_O) δ 4.5 ppm (s) ^13^C NMR (D_2_O) δ 154.6, δ 43.1. IR cm^–1^ 3355 (m, broad), 1652 (m, sharp), 1622 (m, sharp), 1326 (s, sharp).

S3. Synthesis of 4,5-diamino-2-methyl-2H-tetrazol-4-ium perchlorate (**3**).

In a plastic beaker, 100 mg of **1** are dissolved in ~10 mL of water. Next, one equivalent of silver perchlorate (138 mg) is dissolved in water and added dropwise to the stirred solution of **1**, whereupon a white precipitate forms immediately. After stirring for one hour, the white precipitate is separated by filtration, and the filtrate is collected in a plastic beaker and dried under an airline. Crystals suitable for single crystal X-ray diffraction are obtained by diffusion of Et_2_O into a solution in MeOH. Yield: 142 mg (99.5%). ^1^H NMR (D_2_O) δ 4.3 ppm (s), ^13^C NMR (D_2_O) δ 157.3, δ 43.0 ppm. IR (cm^– 1^) 621 (vs), 1050 (vs, broad), 1397 (m) 1622 (m), 1680 (m), 3338 (m, broad).

S4. Synthesis of 4,5-diamino-2-methyl-2H-tetrazol-4-ium 5-nitrotetrazolate-2-*N*-oxide (**4**).

In a plastic beaker, 100 mg of **1** is dissolved in ~10 mL of water. Next, one equivalent of silver 5-nitrotetrazolate-2-*N*-oxide (158 mg) is prepared according to literature^2^ and added to the solution of **1**. After addition, the beaker is covered with aluminum foil and stirred for sixteen hours, whereupon the white precipitate is separated by filtration and the solution is dried under an airline in a plastic beaker. Crystals suitable for single crystal X-ray diffraction are obtained by slow evaporation from water. Yield: 161 mg (99%). ^1^H NMR (D_2_O) δ 4.3 ppm, ^13^C NMR (D_2_O) δ 165.0, δ 157.3, δ 43.0 ppm. IR (cm^–1^) 3142 (m, broad), 2704 (w, broad), 1677 (s), 1618 (s), 1569 (s), 1312 (vs, broad), 1207 (m), 1039 (m), 868 (w), 824 (w), 753 (w), 723 (w), 626 (m), 516 (m).

S5. Synthesis of 1,1’-azobis-3,3’-dimethyl-5,5’-diaminotetrazol-3,3’-ium dinitrate (**5**).

To a glass test tube, 1 mL of white fuming nitric acid is added, whereupon the test tube is placed in an ice water bath and a stir bar is added. Next, 100 mg of 1,5-diamino-3-methyl-tetrazolium chloride (**1**), is added slowly to the stirred nitric acid. After the addition of **1** is complete, the solution is stirred for 30 minutes. During the stir period, the solution yellowed slightly, and some gas evolution is observed. 30 minutes after addition of **1** is complete, the reaction is poured onto 10 mL of chilled distilled water in a plastic beaker, and the solution is placed under an airline and reduced to dryness overnight. Suitable crystals for single crystal x-ray diffraction were obtained using this method, which appear as orange rods. Yield: 70 mg (30%). ^1^H NMR (D_2_O) δ 4.2 ppm, ^13^C NMR (D_2_O) δ 157.5, δ 40.1 ppm. IR (cm^–1^) 3114 (w, broad), 1669, (vs), 1412 (s), 1284 (s), 1284 (vs), 1245 (s), 1169 (s), 1106 (s), 985 (w), 923 (w), 875 (w), 823 (w), 778 (w), 646 (w), 594 (w), 486 (m, broad).

S6. Synthesis of 5-amino-2-methyl-4-nitriminotetrazole (**6**).

In a glass test tube equipped with a magnetic stir bar, 1 mL of white fuming nitric acid is added, whereupon the test tube is chilled to 0 °C with an ice water bath. To this, 100 mg of **1** is added over 5 minutes to the stirred solution. After the addition of **1** is complete, the reaction is stirred for 30 minutes at 0 °C, whereupon the crude reaction mixture is added dropwise to 30 mL of anhydrous Et_2_O. During the addition, a bright yellow precipitate forms, which is filtered and collected within a plastic Büchner funnel. The precipitate (65 mg) is dried, then suspended in 10 mL of absolute ethanol, whereupon one equivalent of NaOEt is added dropwise from a 1 M NaOEt solution at 0 °C. During the addition, the solution immediately changes from bright yellow to orange. After the addition of one equivalent of ethanolic NaOEt, the solution is stirred for one hour, then the inorganic precipitate is removed by filtration and the ethanol is dried under an airline to yield 16 mg (15.1%) of **6**. ^1^H NMR (DMSO *d*-6) δ 4.3 (s), ^13^C NMR (DMSO *d*-6) δ 155.6, δ 43.0 ppm. IR (cm^–1^) 3375 (w, broad), 3111 (w, broad), 1662 (s, sharp), 1418 (m), 1274 (s, strong), 1241 (s, strong). HRMS (M+Na) calculated: 182.03969, found 182.03988.

References:

46. C. Hamdouchi, P. Maiti, A. R. Miller, World Intellectual Property Organization PCT/US2015/010277. July 16, 2015.

47.	Bruker, *SAINT*, *V8.40B*, Bruker AXS Inc., Madison, Wisconsin, USA.

48.	L. Krause, R. Herbst-Irmer, G. M. Sheldrick, D. Stalke, *J. Appl. Cryst.* **2015**, *48*, 3–10, doi:10.1107/S1600576714022985.

49.	G. M. Sheldrick, *Acta Cryst.* **2015**, *A71*, 3–8, doi:10.1107/S2053273314026370.

50.	G. M. Sheldrick, *Acta Cryst.* **2015**, *C71*, 3–8, doi:10.1107/S2053229614024218.

51.	C. R. Groom, I. J. Bruno, M. P. Lightfoot, S. C. Ward, *Acta Cryst.* **2016**, *B72*, 171–179, doi:10.1107/S2052520616003954.

52. D. Kratzert, *FinalCif*, *V154*, https://dkratzert.de/finalcif.html.

Crystallographic Details

Structure Tables for **1:**

Table 1. Crystal data and structure refinement for 1

| CCDC number | 2483220 |
| --- | --- |
| Empirical formula | C_2_H_7_ClN_6_ |
| Formula weight | 150.59 |
| Temperature [K] | 150(2) |
| Crystal system | monoclinic |
| Space group (number) | $P2_{1}/c$ (14) |
| *a* [Å] | 7.2229(2) |
| *b* [Å] | 8.6735(2) |
| *c* [Å] | 11.0267(3) |
| α [°] | 90 |
| β [°] | 108.1986(10) |
| γ [°] | 90 |
| Volume [Å^3^] | 656.24(3) |
| *Z* | 4 |
| *ρ*_calc_ [gcm^−3^] | 1.524 |
| *μ* [mm^−1^] | 0.502 |
| *F*(000) | 312 |
| Crystal size [mm^3^] | 0.150×0.220×0.230 |
| Crystal colour | colourless |
| Crystal shape | plate |
| Radiation | Mo*K_α_* (λ=0.71073 Å) |
| 2θ range [°] | 5.94 to 66.33 (0.65 Å) |
| Index ranges | −11 ≤ h ≤ 11 −13 ≤ k ≤ 13 −16 ≤ l ≤ 16 |
| Reflections collected | 19474 |
| Independent reflections | 2501  *R*_int_ = 0.0333 *R*_sigma_ = 0.0185 |
| Completeness to  θ = 25.242° | 100.0 % |
| Data / Restraints / Parameters | 2501 / 0 / 95 |
| Absorption correction T_min_/T_max_ (method) | 0.6915 / 0.7465  (multi-scan) |
| Goodness-of-fit on *F*^2^ | 1.057 |
| Final *R* indexes  [*I*≥2σ(*I*)] | *R*_1_ = 0.0254 w*R*_2_ = 0.0678 |
| Final *R* indexes  [all data] | *R*_1_ = 0.0303 w*R*_2_ = 0.0701 |
| Largest peak/hole [eÅ^−3^] | 0.36/−0.28 |

Table 2. Atomic coordinates and *U_eq_* [Å^2^] for 1

| **Atom** | ***x*** | ***y*** | ***z*** | ***U*_eq_** |
| --- | --- | --- | --- | --- |
| Cl1 | 0.77428(3) | 0.14748(2) | 0.61726(2) | 0.02115(6) |
| N1 | 0.82482(10) | 0.72988(8) | 0.49204(7) | 0.01706(13) |
| N2 | 0.71760(11) | 0.76977(9) | 0.37303(7) | 0.01943(14) |
| N3 | 0.61820(10) | 0.64648(8) | 0.33226(7) | 0.01858(14) |
| N4 | 0.64980(11) | 0.52654(9) | 0.41327(7) | 0.01997(14) |
| N5 | 0.96390(12) | 0.82359(9) | 0.57369(8) | 0.02179(15) |
| H5A | 0.904(2) | 0.9068(18) | 0.5852(14) | 0.033 |
| H5B | 1.055(2) | 0.8409(15) | 0.5353(14) | 0.033 |
| N6 | 0.87492(13) | 0.50987(9) | 0.62528(8) | 0.02390(16) |
| H6A | 0.851(2) | 0.4175(19) | 0.6312(14) | 0.036 |
| H6B | 0.962(2) | 0.5585(17) | 0.6842(14) | 0.036 |
| C1 | 0.78641(12) | 0.58198(9) | 0.51663(8) | 0.01721(14) |
| C2 | 0.47779(14) | 0.63877(11) | 0.20389(9) | 0.02417(17) |
| H2A | 0.468673 | 0.739843 | 0.162715 | 0.036 |
| H2B | 0.349698 | 0.609460 | 0.209538 | 0.036 |
| H2C | 0.520921 | 0.561828 | 0.153422 | 0.036 |

*U*_eq_ is defined as 1/3 of the trace of the orthogonalized *U_ij_* tensor.

Table 3. Anisotropic displacement parameters [Å^2^] for 1. The anisotropic displacement factor exponent takes the form: −2π^2^[ *h*^2^*(a*)*^2^*U*_11_ *+ k*^2^*(b*)*^2^*U*_22_ + … + *2hka*b*U*_12_ ]

| **Atom** | ***U*_11_** | ***U*_22_** | ***U*_33_** | ***U*_23_** | ***U*_13_** | ***U*_12_** |
| --- | --- | --- | --- | --- | --- | --- |
| Cl1 | 0.02498(10) | 0.01701(10) | 0.02046(10) | −0.00127(6) | 0.00562(7) | −0.00213(7) |
| N1 | 0.0188(3) | 0.0144(3) | 0.0163(3) | 0.0016(2) | 0.0031(2) | −0.0006(2) |
| N2 | 0.0204(3) | 0.0182(3) | 0.0179(3) | 0.0021(2) | 0.0035(2) | 0.0025(2) |
| N3 | 0.0182(3) | 0.0191(3) | 0.0171(3) | 0.0001(2) | 0.0036(2) | 0.0025(2) |
| N4 | 0.0218(3) | 0.0177(3) | 0.0186(3) | 0.0000(2) | 0.0038(3) | −0.0015(3) |
| N5 | 0.0247(3) | 0.0163(3) | 0.0210(3) | −0.0001(3) | 0.0023(3) | −0.0047(3) |
| N6 | 0.0319(4) | 0.0164(3) | 0.0185(3) | 0.0036(3) | 0.0007(3) | −0.0054(3) |
| C1 | 0.0196(3) | 0.0145(3) | 0.0177(3) | 0.0000(3) | 0.0060(3) | −0.0006(3) |
| C2 | 0.0222(4) | 0.0285(4) | 0.0173(4) | −0.0022(3) | −0.0003(3) | 0.0044(3) |

Table 4. Bond lengths and angles for 1

| **Atom–Atom** | **Length [Å]** |
| --- | --- |
| N1–N2 | 1.3441(10) |
| N1–C1 | 1.3576(11) |
| N1–N5 | 1.3841(10) |
| N2–N3 | 1.2882(10) |
| N3–N4 | 1.3434(10) |
| N3–C2 | 1.4629(11) |
| N4–C1 | 1.3422(11) |
| N5–H5A | 0.869(15) |
| N5–H5B | 0.897(16) |
| N6–C1 | 1.3254(11) |
| N6–H6A | 0.827(16) |
| N6–H6B | 0.861(15) |
| C2–H2A | 0.9800 |
| C2–H2B | 0.9800 |
| C2–H2C | 0.9800 |
|  |  |
| **Atom–Atom–Atom** | **Angle [°]** |
| N2–N1–C1 | 110.30(7) |
| N2–N1–N5 | 124.32(7) |
| C1–N1–N5 | 125.24(7) |
| N3–N2–N1 | 102.44(7) |
| N2–N3–N4 | 116.76(7) |
| N2–N3–C2 | 121.20(7) |
| N4–N3–C2 | 122.03(7) |
| C1–N4–N3 | 102.38(7) |
| N1–N5–H5A | 106.9(10) |
| N1–N5–H5B | 106.2(9) |
| H5A–N5–H5B | 113.4(13) |
| C1–N6–H6A | 118.3(10) |
| C1–N6–H6B | 118.9(10) |
| H6A–N6–H6B | 122.6(14) |
| N6–C1–N4 | 128.14(8) |
| N6–C1–N1 | 123.75(8) |
| N4–C1–N1 | 108.10(7) |
| N3–C2–H2A | 109.5 |
| N3–C2–H2B | 109.5 |
| H2A–C2–H2B | 109.5 |
| N3–C2–H2C | 109.5 |
| H2A–C2–H2C | 109.5 |
| H2B–C2–H2C | 109.5 |

Table 5. Torsion angles for 1

| **Atom–Atom–Atom–Atom** | **Torsion Angle [°]** |
| --- | --- |
| C1–N1–N2–N3 | 0.95(9) |
| N5–N1–N2–N3 | 176.80(8) |
| N1–N2–N3–N4 | −0.33(9) |
| N1–N2–N3–C2 | 179.56(7) |
| N2–N3–N4–C1 | −0.41(9) |
| C2–N3–N4–C1 | 179.71(8) |
| N3–N4–C1–N6 | −178.23(9) |
| N3–N4–C1–N1 | 0.97(9) |
| N2–N1–C1–N6 | 177.98(8) |
| N5–N1–C1–N6 | 2.18(14) |
| N2–N1–C1–N4 | −1.26(9) |
| N5–N1–C1–N4 | −177.06(8) |

Table 6. Hydrogen bonds for 1

| **D–H⋯A [Å]** | **d(D–H) [Å]** | **d(H⋯A) [Å]** | **d(D⋯A) [Å]** | **<(DHA) [°]** |
| --- | --- | --- | --- | --- |
| N5–H5A⋯Cl1^#1^ | 0.869(15) | 2.361(16) | 3.2263(9) | 173.7(13) |
| N5–H5B⋯Cl1^#2^ | 0.897(16) | 2.380(16) | 3.2495(9) | 163.3(13) |
| N6–H6A⋯Cl1 | 0.827(16) | 2.400(16) | 3.2211(8) | 172.1(14) |
| N6–H6B⋯Cl1^#3^ | 0.861(15) | 2.532(15) | 3.3781(9) | 167.8(13) |
| C2–H2A⋯N4^#4^ | 0.98 | 2.68 | 3.6176(12) | 160.8 |
| C2–H2C⋯Cl1^#5^ | 0.98 | 2.69 | 3.5973(10) | 153.4 |

Symmetry transformations used to generate equivalent atoms:

#1: +X, 1+Y, +Z; #2: 2-X, 1-Y, 1-Z; #3: 2-X, 0.5+Y, 1.5-Z; #4: 1-X, 0.5+Y, 0.5-Z; #5: +X, 0.5-Y, -0.5+Z;

Structure Tables for **2:**

Table 7. Crystal data and structure refinement for 2

| CCDC number | 2483215 |
| --- | --- |
| Empirical formula | C_2_H_7_N_7_O_3_ |
| Formula weight | 177.15 |
| Temperature [K] | 275(2) |
| Crystal system | monoclinic |
| Space group (number) | $\mathrm{Cc}$ (9) |
| *a* [Å] | 8.5118(4) |
| *b* [Å] | 7.0725(3) |
| *c* [Å] | 12.7541(5) |
| α [°] | 90 |
| β [°] | 96.786(2) |
| γ [°] | 90 |
| Volume [Å^3^] | 762.41(6) |
| *Z* | 4 |
| *ρ*_calc_ [gcm^−3^] | 1.543 |
| *μ* [mm^−1^] | 0.137 |
| *F*(000) | 368 |
| Crystal size [mm^3^] | 0.082×0.096×0.279 |
| Crystal colour | colourless |
| Crystal shape | plate |
| Radiation | Mo*K_α_* (λ=0.71073 Å) |
| 2θ range [°] | 6.43 to 56.62 (0.75 Å) |
| Index ranges | −11 ≤ h ≤ 11 −9 ≤ k ≤ 9 −16 ≤ l ≤ 16 |
| Reflections collected | 8433 |
| Independent reflections | 1893  *R*_int_ = 0.0233 *R*_sigma_ = 0.0215 |
| Completeness to  θ = 25.242° | 100.0 % |
| Data / Restraints / Parameters | 1893 / 6 / 117 |
| Absorption correction T_min_/T_max_ (method) | 0.7013 / 0.7457  (multi-scan) |
| Goodness-of-fit on *F*^2^ | 1.081 |
| Final *R* indexes  [*I*≥2σ(*I*)] | *R*_1_ = 0.0319 w*R*_2_ = 0.0808 |
| Final *R* indexes  [all data] | *R*_1_ = 0.0359 w*R*_2_ = 0.0853 |
| Largest peak/hole [eÅ^−3^] | 0.15/−0.20 |
| Flack X parameter | -1(2) |

Refinement details for 2

Refined as a 1:1 2-component inversion twin. The hydrogens on C2 and N6 were automatically placed. The hydrogens on N5 were placed by Q peaks and their geometries were restrained according to average data based on the CCSD. The positions were refined to hydrogen bond to the nearest nitrate oxygens.

Table 8. Atomic coordinates and *U_eq_* [Å^2^] for 2

| **Atom** | ***x*** | ***y*** | ***z*** | ***U*_eq_** |
| --- | --- | --- | --- | --- |
| O1 | 0.8738(2) | 0.6012(3) | 0.47449(13) | 0.0641(5) |
| O2 | 0.8382(3) | 0.7053(3) | 0.31484(16) | 0.0724(5) |
| O3 | 0.8076(3) | 0.4156(3) | 0.3427(2) | 0.0854(8) |
| N1 | 0.4109(2) | 0.4347(3) | 0.68339(14) | 0.0452(4) |
| N2 | 0.2665(2) | 0.4668(3) | 0.63239(15) | 0.0436(4) |
| N3 | 0.2602(2) | 0.5172(3) | 0.53509(15) | 0.0469(4) |
| N4 | 0.4124(2) | 0.5228(2) | 0.52040(13) | 0.0394(4) |
| N5 | 0.4678(3) | 0.5638(3) | 0.42508(15) | 0.0540(5) |
| H5A | 0.418(4) | 0.672(3) | 0.399(3) | 0.081 |
| H5B | 0.429(5) | 0.456(3) | 0.381(3) | 0.081 |
| N6 | 0.6604(2) | 0.4597(3) | 0.61795(16) | 0.0567(5) |
| H6A | 0.712524 | 0.426095 | 0.676890 | 0.068 |
| H6B | 0.709736 | 0.486083 | 0.564732 | 0.068 |
| N7 | 0.8408(2) | 0.5730(3) | 0.37899(15) | 0.0457(4) |
| C1 | 0.5046(3) | 0.4706(3) | 0.60918(16) | 0.0391(4) |
| C2 | 0.1252(3) | 0.4514(4) | 0.6864(2) | 0.0588(6) |
| H2A | 0.033027 | 0.448035 | 0.635189 | 0.088 |
| H2B | 0.119105 | 0.558504 | 0.731986 | 0.088 |
| H2C | 0.130444 | 0.337465 | 0.727611 | 0.088 |

*U*_eq_ is defined as 1/3 of the trace of the orthogonalized *U_ij_* tensor.

Table 9. Anisotropic displacement parameters [Å^2^] for 2. The anisotropic displacement factor exponent takes the form: −2π^2^[ *h*^2^*(a*)*^2^*U*_11_ *+ k*^2^*(b*)*^2^*U*_22_ + … + *2hka*b*U*_12_ ]

| **Atom** | ***U*_11_** | ***U*_22_** | ***U*_33_** | ***U*_23_** | ***U*_13_** | ***U*_12_** |
| --- | --- | --- | --- | --- | --- | --- |
| O1 | 0.0544(9) | 0.0966(14) | 0.0393(8) | 0.0010(9) | −0.0029(7) | −0.0069(10) |
| O2 | 0.0903(14) | 0.0688(11) | 0.0567(10) | 0.0164(9) | 0.0031(9) | −0.0048(10) |
| O3 | 0.107(2) | 0.0539(10) | 0.0881(16) | −0.0119(10) | −0.0186(14) | 0.0103(10) |
| N1 | 0.0433(10) | 0.0584(10) | 0.0334(8) | 0.0019(7) | 0.0023(7) | 0.0024(8) |
| N2 | 0.0412(9) | 0.0484(9) | 0.0411(9) | −0.0015(7) | 0.0050(7) | 0.0011(7) |
| N3 | 0.0442(9) | 0.0543(10) | 0.0409(10) | 0.0022(8) | 0.0001(7) | 0.0049(8) |
| N4 | 0.0424(9) | 0.0436(8) | 0.0313(7) | 0.0007(7) | 0.0007(7) | 0.0035(7) |
| N5 | 0.0538(12) | 0.0750(14) | 0.0332(9) | 0.0103(9) | 0.0055(8) | 0.0054(10) |
| N6 | 0.0416(10) | 0.0943(16) | 0.0335(9) | 0.0080(9) | 0.0017(7) | 0.0043(10) |
| N7 | 0.0405(9) | 0.0544(10) | 0.0419(9) | 0.0017(7) | 0.0034(7) | 0.0045(7) |
| C1 | 0.0429(9) | 0.0439(9) | 0.0295(8) | −0.0026(7) | 0.0002(7) | 0.0014(8) |
| C2 | 0.0458(12) | 0.0730(16) | 0.0593(14) | 0.0042(11) | 0.0136(10) | −0.0020(10) |

Table 10. Bond lengths and angles for 2

| **Atom–Atom** | **Length [Å]** |
| --- | --- |
| O1–N7 | 1.234(2) |
| O2–N7 | 1.241(3) |
| O3–N7 | 1.226(3) |
| N1–C1 | 1.333(3) |
| N1–N2 | 1.340(3) |
| N2–N3 | 1.286(3) |
| N2–C2 | 1.459(3) |
| N3–N4 | 1.332(3) |
| N4–C1 | 1.351(3) |
| N4–N5 | 1.385(3) |
| N5–H5A | 0.919(13) |
| N5–H5B | 0.979(15) |
| N6–C1 | 1.319(3) |
| N6–H6A | 0.8600 |
| N6–H6B | 0.8600 |
| C2–H2A | 0.9600 |
| C2–H2B | 0.9600 |
| C2–H2C | 0.9600 |
|  |  |
| **Atom–Atom–Atom** | **Angle [°]** |
| C1–N1–N2 | 102.41(17) |
| N3–N2–N1 | 116.54(18) |
| N3–N2–C2 | 122.2(2) |
| N1–N2–C2 | 121.2(2) |
| N2–N3–N4 | 102.32(17) |
| N3–N4–C1 | 110.64(16) |
| N3–N4–N5 | 124.56(18) |
| C1–N4–N5 | 124.66(18) |
| N4–N5–H5A | 108(2) |
| N4–N5–H5B | 102(2) |
| H5A–N5–H5B | 110(4) |
| C1–N6–H6A | 120.0 |
| C1–N6–H6B | 120.0 |
| H6A–N6–H6B | 120.0 |
| O3–N7–O1 | 122.3(2) |
| O3–N7–O2 | 116.8(2) |
| O1–N7–O2 | 120.9(2) |
| N6–C1–N1 | 127.50(19) |
| N6–C1–N4 | 124.44(19) |
| N1–C1–N4 | 108.06(18) |
| N2–C2–H2A | 109.5 |
| N2–C2–H2B | 109.5 |
| H2A–C2–H2B | 109.5 |
| N2–C2–H2C | 109.5 |
| H2A–C2–H2C | 109.5 |
| H2B–C2–H2C | 109.5 |

Table 11. Torsion angles for 2

| **Atom–Atom–Atom–Atom** | **Torsion Angle [°]** |
| --- | --- |
| C1–N1–N2–N3 | 0.4(2) |
| C1–N1–N2–C2 | −177.5(2) |
| N1–N2–N3–N4 | −1.2(2) |
| C2–N2–N3–N4 | 176.6(2) |
| N2–N3–N4–C1 | 1.6(2) |
| N2–N3–N4–N5 | 177.4(2) |
| N2–N1–C1–N6 | −179.7(2) |
| N2–N1–C1–N4 | 0.6(2) |
| N3–N4–C1–N6 | 178.9(2) |
| N5–N4–C1–N6 | 3.1(3) |
| N3–N4–C1–N1 | −1.4(2) |
| N5–N4–C1–N1 | −177.3(2) |

Table 12. Hydrogen bonds for 2

| **D–H⋯A [Å]** | **d(D–H) [Å]** | **d(H⋯A) [Å]** | **d(D⋯A) [Å]** | **<(DHA) [°]** |
| --- | --- | --- | --- | --- |
| N5–H5A⋯O3^#1^ | 0.919(13) | 2.050(13) | 2.969(3) | 179(3) |
| N5–H5B⋯O2^#2^ | 0.979(15) | 2.076(13) | 3.042(3) | 169(4) |
| N5–H5B⋯N1^#3^ | 0.979(15) | 2.62(4) | 3.063(3) | 107(3) |
| C2–H2A⋯O1^#4^ | 0.96 | 2.56 | 3.412(4) | 147.9 |
| C2–H2B⋯O2^#5^ | 0.96 | 2.63 | 3.341(4) | 131.1 |

Symmetry transformations used to generate equivalent atoms:

#1: -0.5+X, 0.5+Y, +Z; #2: -0.5+X, -0.5+Y, +Z; #3: +X, 1-Y, -0.5+Z; #4: -1+X, +Y, +Z; #5: -0.5+X, 1.5-Y, 0.5+Z;

Structure Tables for **3**:

Table 13. Crystal data and structure refinement for 3

| CCDC number | 2483218 |
| --- | --- |
| Empirical formula | C_2_H_7_ClN_6_O_4_ |
| Formula weight | 214.59 |
| Temperature [K] | 299(2) |
| Crystal system | orthorhombic |
| Space group (number) | $\mathrm{Pbca}$ (61) |
| *a* [Å] | 8.1446(5) |
| *b* [Å] | 10.1645(6) |
| *c* [Å] | 20.5899(11) |
| α [°] | 90 |
| β [°] | 90 |
| γ [°] | 90 |
| Volume [Å^3^] | 1704.55(17) |
| *Z* | 8 |
| *ρ*_calc_ [gcm^−3^] | 1.672 |
| *μ* [mm^−1^] | 4.073 |
| *F*(000) | 880 |
| Crystal size [mm^3^] | 0.024×0.473×0.520 |
| Crystal colour | colourless |
| Crystal shape | plate |
| Radiation | Cu*K_α_* (λ=1.54184 Å) |
| 2θ range [°] | 8.59 to 159.83 (0.78 Å) |
| Index ranges | −8 ≤ h ≤ 9 −11 ≤ k ≤ 11 −26 ≤ l ≤ 26 |
| Reflections collected | 9452 |
| Independent reflections | 1765  *R*_int_ = 0.0580 *R*_sigma_ = 0.0406 |
| Completeness to  θ = 67.684° | 98.7 % |
| Data / Restraints / Parameters | 1765 / 823 / 265 |
| Absorption correction T_min_/T_max_ (method) | 0.4240 / 0.7543  (multi-scan) |
| Goodness-of-fit on *F*^2^ | 1.069 |
| Final *R* indexes  [*I*≥2σ(*I*)] | *R*_1_ = 0.0539 w*R*_2_ = 0.1582 |
| Final *R* indexes  [all data] | *R*_1_ = 0.0574 w*R*_2_ = 0.1624 |
| Largest peak/hole [eÅ^−3^] | 0.42/−0.26 |
| Extinction coefficient | 0.0024(6) |

Refinement details for 3

The perchlorate anion is highly disordered by rotation. Four moieties needed to be refined to account for all orientations resolved in the measured electron density. The four disordered moieties were restrained to have similar geometries. For the template perchlorate, all Cl-O distances were restrained to be similar. Uij components of ADPs for disordered atoms closer to each other than 2.0 Angstrom were restrained to be similar. The ADP of least occupied O2D and most occupied O2B (atoms are in nearly identical positions) were constrained to be identical. Subject to these conditions the occupancy ratio refined to 0.255(3) to 0.388(3) to 0.223(3) to 0.134(3).

Table 14. Atomic coordinates and *U_eq_* [Å^2^] for 3

| **Atom** | ***x*** | ***y*** | ***z*** | ***U*_eq_** |
| --- | --- | --- | --- | --- |
| N1 | 0.8342(2) | 0.30302(18) | 0.65092(7) | 0.0589(5) |
| N2 | 0.8038(2) | 0.25642(17) | 0.59156(7) | 0.0557(5) |
| N3 | 0.6884(2) | 0.17170(17) | 0.58689(7) | 0.0577(5) |
| N4 | 0.6370(2) | 0.16025(16) | 0.64809(7) | 0.0538(4) |
| N5 | 0.6984(3) | 0.2513(2) | 0.75034(9) | 0.0755(6) |
| H5A | 0.761(5) | 0.311(4) | 0.7704(18) | 0.091 |
| H5B | 0.627(5) | 0.194(4) | 0.772(2) | 0.091 |
| N6 | 0.5062(3) | 0.0831(2) | 0.66787(11) | 0.0745(6) |
| H6A | 0.414(5) | 0.105(4) | 0.6404(19) | 0.089 |
| H6B | 0.528(5) | −0.005(5) | 0.6641(19) | 0.089 |
| C1 | 0.7244(2) | 0.24018(19) | 0.68735(8) | 0.0524(5) |
| C2 | 0.8965(3) | 0.3016(3) | 0.53488(11) | 0.0812(7) |
| H2A | 0.896551 | 0.234092 | 0.502285 | 0.122 |
| H2B | 1.007380 | 0.320801 | 0.547482 | 0.122 |
| H2C | 0.846182 | 0.379639 | 0.517692 | 0.122 |
| Cl1 | 0.4157(17) | 0.4786(12) | 0.6202(6) | 0.056(2) |
| O1 | 0.5055(17) | 0.5242(14) | 0.6739(6) | 0.094(3) |
| O2 | 0.531(2) | 0.4286(18) | 0.5741(9) | 0.086(5) |
| O3 | 0.321(3) | 0.3672(16) | 0.6326(8) | 0.095(4) |
| O4 | 0.323(3) | 0.5848(17) | 0.5944(8) | 0.068(4) |
| Cl1B | 0.4007(10) | 0.4766(8) | 0.6174(2) | 0.0472(15) |
| O1B | 0.4446(15) | 0.4734(11) | 0.6848(3) | 0.094(2) |
| O2B | 0.539(2) | 0.4655(18) | 0.5770(9) | 0.083(3) |
| O3B | 0.2823(15) | 0.3798(12) | 0.6055(6) | 0.103(3) |
| O4B | 0.3403(17) | 0.6083(10) | 0.6117(6) | 0.079(3) |
| Cl1C | 0.3925(19) | 0.4676(16) | 0.6219(7) | 0.059(3) |
| O1C | 0.362(3) | 0.4855(19) | 0.6881(6) | 0.109(4) |
| O2C | 0.5614(16) | 0.4728(15) | 0.6070(9) | 0.087(3) |
| O3C | 0.336(2) | 0.3367(14) | 0.6113(10) | 0.089(4) |
| O4C | 0.290(2) | 0.550(2) | 0.5838(10) | 0.079(5) |
| Cl1D | 0.406(2) | 0.4699(15) | 0.6139(9) | 0.061(4) |
| O1D | 0.420(3) | 0.5928(18) | 0.6446(11) | 0.097(4) |
| O2D | 0.545(4) | 0.453(5) | 0.573(2) | 0.083(3) |
| O3D | 0.408(2) | 0.3681(18) | 0.6603(11) | 0.095(4) |
| O4D | 0.261(2) | 0.472(3) | 0.5762(10) | 0.091(4) |

*U*_eq_ is defined as 1/3 of the trace of the orthogonalized *U_ij_* tensor.

Table 15. Anisotropic displacement parameters [Å^2^] for 3. The anisotropic displacement factor exponent takes the form: −2π^2^[ *h*^2^*(a*)*^2^*U*_11_ *+ k*^2^*(b*)*^2^*U*_22_ + … + *2hka*b*U*_12_ ]

| **Atom** | ***U*_11_** | ***U*_22_** | ***U*_33_** | ***U*_23_** | ***U*_13_** | ***U*_12_** |
| --- | --- | --- | --- | --- | --- | --- |
| N1 | 0.0566(9) | 0.0666(10) | 0.0537(8) | −0.0044(6) | −0.0050(6) | −0.0012(7) |
| N2 | 0.0536(9) | 0.0647(10) | 0.0487(7) | −0.0028(6) | −0.0017(6) | 0.0087(6) |
| N3 | 0.0570(9) | 0.0607(10) | 0.0555(8) | −0.0110(6) | −0.0069(6) | 0.0114(6) |
| N4 | 0.0482(8) | 0.0537(9) | 0.0596(8) | −0.0017(5) | −0.0046(6) | 0.0063(6) |
| N5 | 0.0852(12) | 0.0934(14) | 0.0477(8) | 0.0017(8) | −0.0046(7) | 0.0040(11) |
| N6 | 0.0595(11) | 0.0707(13) | 0.0932(13) | 0.0038(9) | 0.0020(9) | −0.0060(8) |
| C1 | 0.0499(9) | 0.0579(10) | 0.0493(8) | −0.0002(6) | −0.0072(6) | 0.0087(6) |
| C2 | 0.0841(16) | 0.1015(19) | 0.0580(10) | 0.0060(11) | 0.0148(9) | 0.0047(12) |
| Cl1 | 0.058(4) | 0.030(3) | 0.081(5) | 0.008(2) | 0.001(3) | 0.002(2) |
| O1 | 0.096(7) | 0.094(7) | 0.092(6) | −0.023(5) | −0.006(5) | −0.017(5) |
| O2 | 0.084(7) | 0.077(8) | 0.098(7) | −0.024(5) | 0.000(5) | 0.043(6) |
| O3 | 0.113(8) | 0.079(7) | 0.095(8) | 0.020(6) | 0.010(7) | −0.046(6) |
| O4 | 0.089(8) | 0.046(8) | 0.070(7) | 0.003(6) | −0.005(5) | 0.021(7) |
| Cl1B | 0.048(3) | 0.055(3) | 0.0378(13) | 0.0035(10) | 0.0066(11) | 0.0080(15) |
| O1B | 0.101(6) | 0.121(7) | 0.061(3) | −0.001(3) | −0.006(4) | 0.005(5) |
| O2B | 0.076(4) | 0.070(5) | 0.104(5) | −0.006(4) | 0.043(4) | 0.003(3) |
| O3B | 0.106(6) | 0.082(7) | 0.120(7) | −0.025(6) | 0.007(5) | −0.044(5) |
| O4B | 0.086(5) | 0.045(4) | 0.107(8) | −0.004(4) | 0.021(5) | 0.013(3) |
| Cl1C | 0.062(5) | 0.040(4) | 0.076(6) | 0.004(3) | 0.006(3) | 0.004(3) |
| O1C | 0.129(10) | 0.128(8) | 0.072(6) | −0.007(5) | 0.005(7) | 0.013(9) |
| O2C | 0.051(5) | 0.080(7) | 0.129(9) | 0.004(7) | 0.019(6) | 0.008(4) |
| O3C | 0.113(8) | 0.059(7) | 0.094(7) | 0.005(5) | 0.015(6) | 0.008(6) |
| O4C | 0.080(8) | 0.057(9) | 0.100(9) | 0.021(7) | −0.001(6) | 0.030(7) |
| Cl1D | 0.053(6) | 0.045(6) | 0.084(7) | −0.010(5) | 0.019(5) | 0.005(4) |
| O1D | 0.121(10) | 0.070(9) | 0.101(9) | −0.035(7) | 0.000(8) | −0.007(8) |
| O2D | 0.076(4) | 0.070(5) | 0.104(5) | −0.006(4) | 0.043(4) | 0.003(3) |
| O3D | 0.090(8) | 0.067(8) | 0.129(9) | 0.053(7) | 0.012(7) | 0.017(6) |
| O4D | 0.076(8) | 0.091(11) | 0.106(9) | 0.004(9) | −0.034(7) | 0.004(7) |

Table 16. Bond lengths and angles for 3

| **Atom–Atom** | **Length [Å]** |
| --- | --- |
| N1–C1 | 1.331(3) |
| N1–N2 | 1.334(2) |
| N2–N3 | 1.278(3) |
| N2–C2 | 1.464(3) |
| N3–N4 | 1.333(2) |
| N4–C1 | 1.349(2) |
| N4–N6 | 1.384(3) |
| N5–C1 | 1.319(3) |
| N5–H5A | 0.89(4) |
| N5–H5B | 0.94(4) |
| N6–H6A | 0.96(4) |
| N6–H6B | 0.92(5) |
| C2–H2A | 0.9600 |
| C2–H2B | 0.9600 |
| C2–H2C | 0.9600 |
| Cl1–O3 | 1.395(11) |
| Cl1–O1 | 1.403(12) |
| Cl1–O4 | 1.423(11) |
| Cl1–O2 | 1.428(12) |
| Cl1B–O3B | 1.399(9) |
| Cl1B–O2B | 1.407(9) |
| Cl1B–O4B | 1.431(9) |
| Cl1B–O1B | 1.434(7) |
| Cl1C–O1C | 1.398(13) |
| Cl1C–O2C | 1.410(12) |
| Cl1C–O4C | 1.420(12) |
| Cl1C–O3C | 1.425(13) |
| Cl1D–O1D | 1.406(14) |
| Cl1D–O3D | 1.409(13) |
| Cl1D–O4D | 1.413(13) |
| Cl1D–O2D | 1.424(14) |
|  |  |
| **Atom–Atom–Atom** | **Angle [°]** |
| C1–N1–N2 | 102.77(16) |
| N3–N2–N1 | 116.41(16) |
| N3–N2–C2 | 122.04(18) |
| N1–N2–C2 | 121.5(2) |
| N2–N3–N4 | 102.64(13) |
| N3–N4–C1 | 110.34(16) |
| N3–N4–N6 | 124.72(17) |
| C1–N4–N6 | 124.84(17) |
| C1–N5–H5A | 115(2) |
| C1–N5–H5B | 121(2) |
| H5A–N5–H5B | 124(3) |
| N4–N6–H6A | 107(2) |
| N4–N6–H6B | 112(3) |
| H6A–N6–H6B | 109(4) |
| N5–C1–N1 | 128.4(2) |
| N5–C1–N4 | 123.8(2) |
| N1–C1–N4 | 107.83(16) |
| N2–C2–H2A | 109.5 |
| N2–C2–H2B | 109.5 |
| H2A–C2–H2B | 109.5 |
| N2–C2–H2C | 109.5 |
| H2A–C2–H2C | 109.5 |
| H2B–C2–H2C | 109.5 |
| O3–Cl1–O1 | 114.4(12) |
| O3–Cl1–O4 | 112.9(15) |
| O1–Cl1–O4 | 108.8(12) |
| O3–Cl1–O2 | 101.3(12) |
| O1–Cl1–O2 | 107.4(12) |
| O4–Cl1–O2 | 111.8(13) |
| O3B–Cl1B–O2B | 113.1(10) |
| O3B–Cl1B–O4B | 114.0(9) |
| O2B–Cl1B–O4B | 107.6(10) |
| O3B–Cl1B–O1B | 109.0(8) |
| O2B–Cl1B–O1B | 111.8(11) |
| O4B–Cl1B–O1B | 100.7(8) |
| O1C–Cl1C–O2C | 112.3(14) |
| O1C–Cl1C–O4C | 110.9(14) |
| O2C–Cl1C–O4C | 115.6(13) |
| O1C–Cl1C–O3C | 102.3(13) |
| O2C–Cl1C–O3C | 108.6(13) |
| O4C–Cl1C–O3C | 106.1(14) |
| O1D–Cl1D–O3D | 110.3(17) |
| O1D–Cl1D–O4D | 107.9(16) |
| O3D–Cl1D–O4D | 113.1(17) |
| O1D–Cl1D–O2D | 108(2) |
| O3D–Cl1D–O2D | 108(2) |
| O4D–Cl1D–O2D | 110(2) |

Table 17. Torsion angles for 3

| **Atom–Atom–Atom–Atom** | **Torsion Angle [°]** |
| --- | --- |
| C1–N1–N2–N3 | −0.2(2) |
| C1–N1–N2–C2 | 178.9(2) |
| N1–N2–N3–N4 | 0.0(2) |
| C2–N2–N3–N4 | −179.19(19) |
| N2–N3–N4–C1 | 0.28(19) |
| N2–N3–N4–N6 | 176.85(19) |
| N2–N1–C1–N5 | −178.7(2) |
| N2–N1–C1–N4 | 0.40(18) |
| N3–N4–C1–N5 | 178.69(19) |
| N6–N4–C1–N5 | 2.1(3) |
| N3–N4–C1–N1 | −0.45(19) |
| N6–N4–C1–N1 | −177.00(18) |

Table 18. Hydrogen bonds for 3

| **D–H⋯A [Å]** | **d(D–H) [Å]** | **d(H⋯A) [Å]** | **d(D⋯A) [Å]** | **<(DHA) [°]** |
| --- | --- | --- | --- | --- |
| N5–H5A⋯O3^a^#1^ | 0.89(4) | 2.13(4) | 2.862(13) | 138(3) |
| N5–H5A⋯O1B^b^#1^ | 0.89(4) | 2.41(5) | 3.302(13) | 175(3) |
| N5–H5A⋯O3B^b^#1^ | 0.89(4) | 2.66(4) | 3.315(13) | 131(3) |
| N5–H5A⋯O1C^c^#1^ | 0.89(4) | 2.13(5) | 3.01(2) | 166(4) |
| N5–H5A⋯O3C^c^#1^ | 0.89(4) | 2.53(4) | 3.182(19) | 131(3) |
| N5–H5A⋯O3D^d^#1^ | 0.89(4) | 1.95(4) | 2.775(15) | 153(4) |
| N5–H5B⋯O1^a^#2^ | 0.94(4) | 2.32(5) | 3.243(13) | 169(3) |
| N5–H5B⋯O1B^b^#2^ | 0.94(4) | 2.48(5) | 3.334(11) | 152(3) |
| N5–H5B⋯O4B^b^#2^ | 0.94(4) | 2.56(4) | 3.206(12) | 126(3) |
| N5–H5B⋯O1C^c^#2^ | 0.94(4) | 2.27(5) | 3.025(17) | 137(3) |
| N5–H5B⋯O1D^d^#2^ | 0.94(4) | 2.04(5) | 2.865(16) | 146(3) |
| N6–H6A⋯O4^a^#3^ | 0.96(4) | 2.16(5) | 3.08(2) | 158(3) |
| N6–H6A⋯O4B^b^#3^ | 0.96(4) | 2.16(4) | 3.061(13) | 155(3) |
| N6–H6A⋯O3C^c | 0.96(4) | 2.51(5) | 3.152(19) | 124(3) |
| N6–H6A⋯O4C^c^#3^ | 0.96(4) | 2.11(5) | 2.99(2) | 151(4) |
| N6–H6A⋯O4D^d^#3^ | 0.96(4) | 2.37(5) | 3.09(2) | 131(3) |
| N6–H6B⋯N1^#4^ | 0.92(5) | 2.26(5) | 3.148(3) | 161(4) |
| C2–H2A⋯O2^a^#5^ | 0.96 | 2.53 | 3.422(19) | 154.4 |
| C2–H2A⋯O3C^c^#5^ | 0.96 | 2.50 | 3.358(17) | 149.4 |
| C2–H2B⋯O3B^b^#6^ | 0.96 | 2.61 | 3.552(11) | 168.1 |
| C2–H2B⋯O4D^d^#6^ | 0.96 | 2.64 | 3.54(2) | 156.2 |
| C2–H2C⋯O4C^c^#7^ | 0.96 | 2.47 | 3.248(15) | 137.9 |
| C2–H2C⋯O4D^d^#7^ | 0.96 | 2.60 | 3.49(2) | 153.6 |

Symmetry transformations used to generate equivalent atoms:

#1: 0.5+X, +Y, 1.5-Z; #2: 1-X, -0.5+Y, 1.5-Z; #3: 0.5-X, -0.5+Y, +Z; #4: 1.5-X, -0.5+Y, +Z; #5: 0.5+X, 0.5-Y, 1-Z; #6: 1+X, +Y, +Z; #7: 1-X, 1-Y, 1-Z;

Structure Tables for **4**:

Table 19. Crystal data and structure refinement for 4

| CCDC number | 2483216 |
| --- | --- |
| Empirical formula | C_3_H_7_N_11_O_3_ |
| Formula weight | 245.20 |
| Temperature [K] | 150(2) |
| Crystal system | monoclinic |
| Space group (number) | $P2_{1}/n$ (14) |
| *a* [Å] | 9.9958(3) |
| *b* [Å] | 6.6699(2) |
| *c* [Å] | 14.5007(5) |
| α [°] | 90 |
| β [°] | 101.2700(10) |
| γ [°] | 90 |
| Volume [Å^3^] | 948.13(5) |
| *Z* | 4 |
| *ρ*_calc_ [gcm^−3^] | 1.718 |
| *μ* [mm^−1^] | 0.148 |
| *F*(000) | 504 |
| Crystal size [mm^3^] | 0.190×0.320×0.430 |
| Crystal colour | light yellow |
| Crystal shape | fragment |
| Radiation | Mo*K_α_* (λ=0.71073 Å) |
| 2θ range [°] | 5.49 to 66.34 (0.65 Å) |
| Index ranges | −15 ≤ h ≤ 15 −10 ≤ k ≤ 10 −19 ≤ l ≤ 22 |
| Reflections collected | 24579 |
| Independent reflections | 3638  *R*_int_ = 0.0243 *R*_sigma_ = 0.0166 |
| Completeness to  θ = 25.242° | 99.9 % |
| Data / Restraints / Parameters | 3638 / 0 / 167 |
| Absorption correction T_min_/T_max_ (method) | 0.6762 / 0.7465  (multi-scan) |
| Goodness-of-fit on *F*^2^ | 1.046 |
| Final *R* indexes  [*I*≥2σ(*I*)] | *R*_1_ = 0.0310 w*R*_2_ = 0.0846 |
| Final *R* indexes  [all data] | *R*_1_ = 0.0341 w*R*_2_ = 0.0868 |
| Largest peak/hole [eÅ^−3^] | 0.42/−0.28 |

Table 20. Atomic coordinates and *U_eq_* [Å^2^] for 4

| **Atom** | ***x*** | ***y*** | ***z*** | ***U*_eq_** |
| --- | --- | --- | --- | --- |
| O1 | 0.41206(6) | 0.78844(9) | 0.69837(4) | 0.01986(11) |
| O2 | 0.79877(6) | 0.67912(10) | 0.52855(4) | 0.02442(12) |
| O3 | 0.66195(7) | 0.66914(11) | 0.39209(4) | 0.02877(14) |
| N1 | 0.58326(6) | 0.73574(9) | 0.61520(4) | 0.01563(11) |
| N2 | 0.45416(6) | 0.77645(9) | 0.62092(4) | 0.01539(11) |
| N3 | 0.37062(6) | 0.79861(11) | 0.53706(4) | 0.02045(12) |
| N4 | 0.44794(7) | 0.76876(10) | 0.47252(4) | 0.01927(12) |
| N5 | 0.68567(7) | 0.69184(9) | 0.47755(4) | 0.01754(11) |
| N6 | 0.42453(6) | 0.47600(9) | 0.86826(4) | 0.01618(11) |
| N7 | 0.53763(6) | 0.58283(9) | 0.86699(4) | 0.01524(11) |
| N8 | 0.62455(6) | 0.50611(9) | 0.82197(4) | 0.01560(11) |
| N9 | 0.56514(6) | 0.33220(9) | 0.79071(4) | 0.01440(11) |
| N10 | 0.62767(6) | 0.18694(10) | 0.74531(4) | 0.01778(12) |
| H10A | 0.6183(12) | 0.2155(18) | 0.6830(8) | 0.021 |
| H10B | 0.7130(12) | 0.1841(18) | 0.7706(8) | 0.021 |
| N11 | 0.36205(6) | 0.15360(10) | 0.80201(5) | 0.01800(11) |
| H11A | 0.3806(11) | 0.0636(18) | 0.7620(8) | 0.022 |
| H11B | 0.2785(12) | 0.1754(18) | 0.8062(8) | 0.022 |
| C1 | 0.57157(7) | 0.73355(10) | 0.52191(5) | 0.01490(12) |
| C2 | 0.44370(6) | 0.31199(10) | 0.81894(4) | 0.01427(11) |
| C3 | 0.56151(8) | 0.77740(11) | 0.91330(5) | 0.01983(13) |
| H3A | 0.612265 | 0.863634 | 0.877510 | 0.030 |
| H3B | 0.614478 | 0.759084 | 0.977130 | 0.030 |
| H3C | 0.473839 | 0.840154 | 0.916356 | 0.030 |

*U*_eq_ is defined as 1/3 of the trace of the orthogonalized *U_ij_* tensor.

Table 21. Anisotropic displacement parameters [Å^2^] for 4. The anisotropic displacement factor exponent takes the form: −2π^2^[ *h*^2^*(a*)*^2^*U*_11_ *+ k*^2^*(b*)*^2^*U*_22_ + … + *2hka*b*U*_12_ ]

| **Atom** | ***U*_11_** | ***U*_22_** | ***U*_33_** | ***U*_23_** | ***U*_13_** | ***U*_12_** |
| --- | --- | --- | --- | --- | --- | --- |
| O1 | 0.0170(2) | 0.0270(3) | 0.0168(2) | −0.00079(19) | 0.00637(18) | −0.00194(19) |
| O2 | 0.0166(2) | 0.0338(3) | 0.0224(3) | −0.0007(2) | 0.00261(19) | 0.0044(2) |
| O3 | 0.0324(3) | 0.0396(3) | 0.0146(2) | −0.0027(2) | 0.0055(2) | 0.0057(3) |
| N1 | 0.0126(2) | 0.0197(3) | 0.0143(2) | 0.00061(19) | 0.00192(18) | 0.00026(18) |
| N2 | 0.0122(2) | 0.0181(2) | 0.0156(2) | 0.00017(19) | 0.00185(18) | −0.00140(18) |
| N3 | 0.0141(3) | 0.0284(3) | 0.0174(3) | 0.0009(2) | −0.0005(2) | 0.0001(2) |
| N4 | 0.0166(3) | 0.0243(3) | 0.0156(2) | 0.0004(2) | −0.00036(19) | 0.0001(2) |
| N5 | 0.0190(3) | 0.0180(3) | 0.0158(2) | 0.00023(19) | 0.0037(2) | 0.0020(2) |
| N6 | 0.0124(2) | 0.0178(2) | 0.0194(3) | 0.0009(2) | 0.00559(18) | 0.00001(18) |
| N7 | 0.0124(2) | 0.0163(2) | 0.0174(2) | 0.00161(19) | 0.00396(18) | 0.00058(18) |
| N8 | 0.0124(2) | 0.0179(2) | 0.0169(2) | 0.00163(19) | 0.00381(18) | 0.00024(18) |
| N9 | 0.0105(2) | 0.0181(2) | 0.0153(2) | 0.00060(19) | 0.00412(17) | 0.00027(18) |
| N10 | 0.0137(2) | 0.0233(3) | 0.0174(3) | −0.0031(2) | 0.00564(19) | 0.0022(2) |
| N11 | 0.0132(2) | 0.0189(3) | 0.0230(3) | −0.0013(2) | 0.0060(2) | −0.00190(19) |
| C1 | 0.0148(3) | 0.0156(3) | 0.0138(3) | 0.0003(2) | 0.0015(2) | 0.0001(2) |
| C2 | 0.0107(2) | 0.0179(3) | 0.0148(3) | 0.0028(2) | 0.00400(19) | 0.00127(19) |
| C3 | 0.0189(3) | 0.0171(3) | 0.0232(3) | −0.0019(2) | 0.0035(2) | −0.0002(2) |

Table 22. Bond lengths and angles for 4

| **Atom–Atom** | **Length [Å]** |
| --- | --- |
| O1–N2 | 1.2765(8) |
| O2–N5 | 1.2269(8) |
| O3–N5 | 1.2248(8) |
| N1–C1 | 1.3350(8) |
| N1–N2 | 1.3370(8) |
| N2–N3 | 1.3420(8) |
| N3–N4 | 1.3400(9) |
| N4–C1 | 1.3226(9) |
| N5–C1 | 1.4417(9) |
| N6–N7 | 1.3395(8) |
| N6–C2 | 1.3410(9) |
| N7–N8 | 1.2902(8) |
| N7–C3 | 1.4592(10) |
| N8–N9 | 1.3410(8) |
| N9–C2 | 1.3614(8) |
| N9–N10 | 1.3870(8) |
| N10–H10A | 0.910(12) |
| N10–H10B | 0.861(12) |
| N11–C2 | 1.3282(9) |
| N11–H11A | 0.879(12) |
| N11–H11B | 0.861(12) |
| C3–H3A | 0.9800 |
| C3–H3B | 0.9800 |
| C3–H3C | 0.9800 |
|  |  |
| **Atom–Atom–Atom** | **Angle [°]** |
| C1–N1–N2 | 99.83(5) |
| O1–N2–N1 | 123.74(6) |
| O1–N2–N3 | 122.44(6) |
| N1–N2–N3 | 113.79(6) |
| N4–N3–N2 | 105.95(6) |
| C1–N4–N3 | 104.69(6) |
| O3–N5–O2 | 125.08(7) |
| O3–N5–C1 | 117.49(6) |
| O2–N5–C1 | 117.42(6) |
| N7–N6–C2 | 102.57(5) |
| N8–N7–N6 | 116.71(6) |
| N8–N7–C3 | 121.32(6) |
| N6–N7–C3 | 121.97(6) |
| N7–N8–N9 | 102.51(5) |
| N8–N9–C2 | 110.24(5) |
| N8–N9–N10 | 123.63(5) |
| C2–N9–N10 | 125.77(6) |
| N9–N10–H10A | 110.9(7) |
| N9–N10–H10B | 108.3(8) |
| H10A–N10–H10B | 109.2(11) |
| C2–N11–H11A | 117.7(8) |
| C2–N11–H11B | 115.0(8) |
| H11A–N11–H11B | 119.6(11) |
| N4–C1–N1 | 115.73(6) |
| N4–C1–N5 | 121.98(6) |
| N1–C1–N5 | 122.27(6) |
| N11–C2–N6 | 126.92(6) |
| N11–C2–N9 | 125.10(6) |
| N6–C2–N9 | 107.96(6) |
| N7–C3–H3A | 109.5 |
| N7–C3–H3B | 109.5 |
| H3A–C3–H3B | 109.5 |
| N7–C3–H3C | 109.5 |
| H3A–C3–H3C | 109.5 |
| H3B–C3–H3C | 109.5 |

Table 23. Torsion angles for 4

| **Atom–Atom–Atom–Atom** | **Torsion Angle [°]** |
| --- | --- |
| C1–N1–N2–O1 | 177.87(6) |
| C1–N1–N2–N3 | −0.40(8) |
| O1–N2–N3–N4 | −177.56(6) |
| N1–N2–N3–N4 | 0.74(8) |
| N2–N3–N4–C1 | −0.72(8) |
| C2–N6–N7–N8 | 0.68(8) |
| C2–N6–N7–C3 | −179.90(6) |
| N6–N7–N8–N9 | −0.37(7) |
| C3–N7–N8–N9 | −179.79(6) |
| N7–N8–N9–C2 | −0.11(7) |
| N7–N8–N9–N10 | −173.60(6) |
| N3–N4–C1–N1 | 0.54(9) |
| N3–N4–C1–N5 | 179.44(6) |
| N2–N1–C1–N4 | −0.10(8) |
| N2–N1–C1–N5 | −179.00(6) |
| O3–N5–C1–N4 | −7.47(10) |
| O2–N5–C1–N4 | 173.50(7) |
| O3–N5–C1–N1 | 171.36(7) |
| O2–N5–C1–N1 | −7.67(10) |
| N7–N6–C2–N11 | 177.55(7) |
| N7–N6–C2–N9 | −0.68(7) |
| N8–N9–C2–N11 | −177.75(6) |
| N10–N9–C2–N11 | −4.44(11) |
| N8–N9–C2–N6 | 0.53(7) |
| N10–N9–C2–N6 | 173.84(6) |

Table 24. Hydrogen bonds for 4

| **D–H⋯A [Å]** | **d(D–H) [Å]** | **d(H⋯A) [Å]** | **d(D⋯A) [Å]** | **<(DHA) [°]** |
| --- | --- | --- | --- | --- |
| N10–H10A⋯N4^#1^ | 0.910(12) | 2.226(12) | 3.1149(9) | 165.5(11) |
| N10–H10B⋯N1^#2^ | 0.861(12) | 2.385(12) | 3.2035(9) | 159.0(10) |
| N10–H10B⋯N8^#2^ | 0.861(12) | 2.589(12) | 3.0798(8) | 117.3(9) |
| N11–H11A⋯O1^#3^ | 0.879(12) | 2.106(12) | 2.9553(9) | 162.4(11) |
| N11–H11B⋯O1^#4^ | 0.861(12) | 2.038(12) | 2.8828(8) | 166.7(11) |
| C3–H3A⋯O2^#5^ | 0.98 | 2.57 | 3.0628(10) | 111.2 |
| C3–H3B⋯N3^#6^ | 0.98 | 2.57 | 3.2950(10) | 131.1 |
| C3–H3C⋯O2^#7^ | 0.98 | 2.62 | 3.3898(10) | 136.1 |

Symmetry transformations used to generate equivalent atoms:

#1: 1-X, 1-Y, 1-Z; #2: 1.5-X, -0.5+Y, 1.5-Z; #3: +X, -1+Y, +Z; #4: 0.5-X, -0.5+Y, 1.5-Z; #5: 1.5-X, 0.5+Y, 1.5-Z; #6: 0.5+X, 1.5-Y, 0.5+Z; #7: -0.5+X, 1.5-Y, 0.5+Z;

Structure Tables for **5**:

Table 25. Crystal data and structure refinement for 5

| CCDC number | 2483217 |
| --- | --- |
| Empirical formula | C_4_H_10_N_14_O_6_ |
| Formula weight | 350.26 |
| Temperature [K] | 150(2) |
| Crystal system | monoclinic |
| Space group (number) | $C2/c$ (15) |
| *a* [Å] | 5.0092(4) |
| *b* [Å] | 22.6877(19) |
| *c* [Å] | 12.1283(10) |
| α [°] | 90 |
| β [°] | 93.080(2) |
| γ [°] | 90 |
| Volume [Å^3^] | 1376.4(2) |
| *Z* | 4 |
| *ρ*_calc_ [gcm^−3^] | 1.690 |
| *μ* [mm^−1^] | 1.340 |
| *F*(000) | 720 |
| Crystal size [mm^3^] | 0.061×0.116×0.358 |
| Crystal colour | colourless |
| Crystal shape | block |
| Radiation | Cu*K_α_* (λ=1.54178 Å) |
| 2θ range [°] | 7.79 to 160.23 (0.78 Å) |
| Index ranges | −6 ≤ h ≤ 6 0 ≤ k ≤ 28 0 ≤ l ≤ 15 |
| Reflections collected | 16407 |
| Independent reflections | 1499  *R*_int_ = 0.0595 *R*_sigma_ = 0.0340 |
| Completeness to  θ = 67.679° | 99.6 % |
| Data / Restraints / Parameters | 1499 / 2 / 121 |
| Absorption correction T_min_/T_max_ (method) | 0.5053 / 0.7543  (multi-scan) |
| Goodness-of-fit on *F*^2^ | 1.054 |
| Final *R* indexes  [*I*≥2σ(*I*)] | *R*_1_ = 0.0395 w*R*_2_ = 0.1044 |
| Final *R* indexes  [all data] | *R*_1_ = 0.0434 w*R*_2_ = 0.1075 |
| Largest peak/hole [eÅ^−3^] | 0.21/−0.26 |
| Extinction coefficient | 0.0023(5) |

Refinement details for 5

The crystal under investigation was found to be non-merohedrally twinned. The orientation matrices for the two components were identified using the program Cell_Now, with the two components being related by a 180 degree rotation around the real/reciprocal axis 0.008 0.000 1.000 and real axis -0.247 -0.001 1.000. The two components were integrated using Saint and corrected for absorption using twinabs, resulting in the following statistics: 9910 data (	1113 unique ) involve domain 1 only, mean I/sigma 61.3	9977 data (	1121 unique ) involve domain 2 only, mean I/sigma 51.1	6571 data (	968 unique ) involve	2 domains, mean I/sigma 76.0 The exact twin matrix identified by the integration program was found to be: -1.00000 0.00008 -0.00001	-0.00169 -1.00000 0.00020	0.26011 0.00005 1.00000 The structure was solved using dual methods with only the non-overlapping reflections of component 1. The structure was refined using the hklf 5 routine with all reflections of component 1 (including the overlapping ones), resulting in a BASF value of 0.402(2). The Rint value given is for all reflections and is based on agreement between observed single and composite intensities and those calculated from refined unique intensities and twin fractions (TWINABS (Sheldrick, 2012)).

Table 26. Atomic coordinates and *U_eq_* [Å^2^] for 5

| **Atom** | ***x*** | ***y*** | ***z*** | ***U*_eq_** |
| --- | --- | --- | --- | --- |
| N1 | 0.6623(3) | 0.15317(7) | −0.01882(12) | 0.0244(4) |
| N2 | 0.8905(3) | 0.15205(6) | 0.04390(13) | 0.0239(3) |
| N3 | 1.0202(3) | 0.10332(6) | 0.05236(12) | 0.0223(3) |
| N4 | 0.8638(3) | 0.06796(6) | −0.01348(11) | 0.0193(3) |
| N5 | 0.8996(3) | 0.00880(6) | −0.02786(11) | 0.0196(3) |
| N6 | 0.4542(3) | 0.07440(7) | −0.12170(12) | 0.0238(3) |
| H6A | 0.328(4) | 0.0969(9) | −0.1542(18) | 0.029 |
| H6B | 0.463(5) | 0.0366(7) | −0.1401(19) | 0.029 |
| C1 | 0.6425(3) | 0.09766(7) | −0.05653(13) | 0.0205(4) |
| C2 | 0.9917(5) | 0.20508(8) | 0.10028(18) | 0.0351(5) |
| H2A | 1.155021 | 0.195492 | 0.144769 | 0.053 |
| H2B | 0.856381 | 0.220197 | 0.148409 | 0.053 |
| H2C | 1.031550 | 0.235112 | 0.045463 | 0.053 |
| O1 | 0.500000 | 0.12733(9) | 0.250000 | 0.0430(6) |
| O2 | 0.6188(3) | 0.04488(7) | 0.17842(12) | 0.0384(4) |
| N7 | 0.500000 | 0.07269(9) | 0.250000 | 0.0267(5) |
| O3 | 0.500000 | 0.38025(8) | 0.250000 | 0.0347(5) |
| O4 | 0.3676(5) | 0.29903(10) | 0.17708(18) | 0.0675(7) |
| N8 | 0.500000 | 0.32443(10) | 0.250000 | 0.0284(5) |

*U*_eq_ is defined as 1/3 of the trace of the orthogonalized *U_ij_* tensor.

Table 27. Anisotropic displacement parameters [Å^2^] for 5. The anisotropic displacement factor exponent takes the form: −2π^2^[ *h*^2^*(a*)*^2^*U*_11_ *+ k*^2^*(b*)*^2^*U*_22_ + … + *2hka*b*U*_12_ ]

| **Atom** | ***U*_11_** | ***U*_22_** | ***U*_33_** | ***U*_23_** | ***U*_13_** | ***U*_12_** |
| --- | --- | --- | --- | --- | --- | --- |
| N1 | 0.0264(8) | 0.0186(7) | 0.0277(7) | 0.0014(5) | −0.0033(6) | 0.0022(6) |
| N2 | 0.0267(7) | 0.0161(7) | 0.0286(7) | 0.0007(5) | −0.0016(6) | 0.0015(6) |
| N3 | 0.0238(7) | 0.0174(6) | 0.0255(7) | −0.0004(5) | −0.0020(6) | −0.0004(6) |
| N4 | 0.0199(7) | 0.0163(6) | 0.0216(6) | 0.0008(5) | −0.0009(5) | 0.0006(5) |
| N5 | 0.0219(7) | 0.0151(7) | 0.0215(7) | 0.0012(5) | 0.0005(5) | 0.0012(5) |
| N6 | 0.0228(7) | 0.0231(7) | 0.0250(7) | 0.0010(6) | −0.0029(6) | 0.0026(6) |
| C1 | 0.0210(8) | 0.0201(8) | 0.0203(7) | 0.0031(6) | 0.0016(6) | 0.0027(7) |
| C2 | 0.0447(12) | 0.0180(8) | 0.0413(10) | −0.0051(7) | −0.0105(10) | −0.0002(9) |
| O1 | 0.0621(15) | 0.0196(9) | 0.0453(12) | 0.000 | −0.0163(11) | 0.000 |
| O2 | 0.0423(9) | 0.0395(8) | 0.0349(7) | −0.0100(6) | 0.0155(7) | −0.0101(7) |
| N7 | 0.0320(12) | 0.0235(10) | 0.0243(10) | 0.000 | −0.0020(9) | 0.000 |
| O3 | 0.0305(10) | 0.0213(9) | 0.0521(12) | 0.000 | 0.0016(9) | 0.000 |
| O4 | 0.0739(16) | 0.0638(12) | 0.0625(12) | −0.0216(10) | −0.0166(11) | −0.0283(11) |
| N8 | 0.0301(13) | 0.0228(10) | 0.0314(11) | 0.000 | −0.0051(9) | 0.000 |

Table 28. Bond lengths and angles for 5

| **Atom–Atom** | **Length [Å]** |
| --- | --- |
| N1–N2 | 1.339(2) |
| N1–C1 | 1.342(2) |
| N2–N3 | 1.284(2) |
| N2–C2 | 1.461(2) |
| N3–N4 | 1.352(2) |
| N4–N5 | 1.3667(18) |
| N4–C1 | 1.376(2) |
| N5–N5^#1^ | 1.247(3) |
| N6–C1 | 1.309(2) |
| N6–H6A | 0.890(16) |
| N6–H6B | 0.888(16) |
| C2–H2A | 0.9800 |
| C2–H2B | 0.9800 |
| C2–H2C | 0.9800 |
| O1–N7 | 1.240(3) |
| O2–N7 | 1.2495(18) |
| O3–N8 | 1.266(3) |
| O4–N8 | 1.221(2) |
|  |  |
| **Atom–Atom–Atom** | **Angle [°]** |
| N2–N1–C1 | 102.76(14) |
| N3–N2–N1 | 118.35(14) |
| N3–N2–C2 | 120.69(16) |
| N1–N2–C2 | 120.95(15) |
| N2–N3–N4 | 100.90(14) |
| N3–N4–N5 | 125.62(14) |
| N3–N4–C1 | 111.29(13) |
| N5–N4–C1 | 122.75(14) |
| N5^#1^–N5–N4 | 110.66(17) |
| C1–N6–H6A | 120.6(15) |
| C1–N6–H6B | 119.7(16) |
| H6A–N6–H6B | 119(2) |
| N6–C1–N1 | 128.46(16) |
| N6–C1–N4 | 124.86(15) |
| N1–C1–N4 | 106.68(15) |
| N2–C2–H2A | 109.5 |
| N2–C2–H2B | 109.5 |
| H2A–C2–H2B | 109.5 |
| N2–C2–H2C | 109.5 |
| H2A–C2–H2C | 109.5 |
| H2B–C2–H2C | 109.5 |
| O1–N7–O2^#2^ | 120.32(11) |
| O1–N7–O2 | 120.32(11) |
| O2^#2^–N7–O2 | 119.4(2) |
| O4–N8–O4^#2^ | 123.7(3) |
| O4–N8–O3 | 118.17(14) |
| O4^#2^–N8–O3 | 118.17(14) |

Symmetry transformations used to generate equivalent atoms:

#1: 2-X, -Y, -Z; #2: 1-X, +Y, 0.5-Z;

Table 29. Torsion angles for 5

| **Atom–Atom–Atom–Atom** | **Torsion Angle [°]** |
| --- | --- |
| C1–N1–N2–N3 | −0.4(2) |
| C1–N1–N2–C2 | 178.86(17) |
| N1–N2–N3–N4 | 1.0(2) |
| C2–N2–N3–N4 | −178.18(16) |
| N2–N3–N4–N5 | −174.75(15) |
| N2–N3–N4–C1 | −1.32(18) |
| N3–N4–N5–N5^#1^ | −0.6(3) |
| C1–N4–N5–N5^#1^ | −173.30(17) |
| N2–N1–C1–N6 | 179.67(17) |
| N2–N1–C1–N4 | −0.52(17) |
| N3–N4–C1–N6 | −178.97(16) |
| N5–N4–C1–N6 | −5.3(3) |
| N3–N4–C1–N1 | 1.20(19) |
| N5–N4–C1–N1 | 174.85(14) |

Symmetry transformations used to generate equivalent atoms:

#1: 2-X, -Y, -Z;

Table 30. Hydrogen bonds for 5

| **D–H⋯A [Å]** | **d(D–H) [Å]** | **d(H⋯A) [Å]** | **d(D⋯A) [Å]** | **<(DHA) [°]** |
| --- | --- | --- | --- | --- |
| N6–H6A⋯O3^#1^ | 0.890(16) | 2.027(17) | 2.8769(17) | 159(2) |
| N6–H6A⋯O4^#1^ | 0.890(16) | 2.564(19) | 3.344(3) | 146.7(19) |
| N6–H6B⋯O2^#2^ | 0.888(16) | 1.945(17) | 2.811(2) | 165(2) |

Symmetry transformations used to generate equivalent atoms:

#1: 0.5-X, 0.5-Y, -Z; #2: 1-X, -Y, -Z;

Structure Tables for **6**:

Table 31. Crystal data and structure refinement for 6

| CCDC number | 2483219 |
| --- | --- |
| Empirical formula | C_2_H_5_N_7_O_2_ |
| Formula weight | 159.13 |
| Temperature [K] | 150(2) |
| Crystal system | monoclinic |
| Space group (number) | $P2_{1}/n$ (14) |
| *a* [Å] | 10.097(3) |
| *b* [Å] | 5.6167(12) |
| *c* [Å] | 11.062(3) |
| α [°] | 90 |
| β [°] | 98.321(8) |
| γ [°] | 90 |
| Volume [Å^3^] | 620.7(3) |
| *Z* | 4 |
| *ρ*_calc_ [gcm^−3^] | 1.703 |
| *μ* [mm^−1^] | 0.147 |
| *F*(000) | 328 |
| Crystal size [mm^3^] | 0.110×0.130×0.390 |
| Crystal colour | light brown |
| Crystal shape | fragment |
| Radiation | Mo*K_α_* (λ=0.71073 Å) |
| 2θ range [°] | 5.91 to 66.35 (0.65 Å) |
| Index ranges | −15 ≤ h ≤ 15 0 ≤ k ≤ 8 0 ≤ l ≤ 17 |
| Reflections collected | 19540 |
| Independent reflections | 2471  *R*_int_ = 0.0642 *R*_sigma_ = 0.0517 |
| Completeness to  θ = 25.242° | 99.8 % |
| Data / Restraints / Parameters | 2471 / 0 / 108 |
| Absorption correction T_min_/T_max_ (method) | 0.5543 / 0.7465  (multi-scan) |
| Goodness-of-fit on *F*^2^ | 1.091 |
| Final *R* indexes  [*I*≥2σ(*I*)] | *R*_1_ = 0.0432 w*R*_2_ = 0.1094 |
| Final *R* indexes  [all data] | *R*_1_ = 0.0470 w*R*_2_ = 0.1133 |
| Largest peak/hole [eÅ^−3^] | 0.37/−0.29 |

Refinement details for 6

The crystal under investigation was found to be non-merohedrally twinned. The orientation matrices for the two components were identified using the program Cell_Now, with the two components being related by a 180 degree rotation around the reciprocal axis (1 0 0). The two components were integrated using Saint and corrected for absorption using twinabs, resulting in the following statistics: 5563 data (1193 unique) involve domain 1 only, mean I/sigma 22.8 5558 data (1192 unique) involve domain 2 only, mean I/sigma 17.0 14070 data (2364 unique) involve 2 domains, mean I/sigma 18.6 4 data (4 unique) involve 3 domains, mean I/sigma 9.0 The exact twin matrix identified by the integration program was found to be: 0.99970 0.00026 0.26596	0.00012 -1.00000 -0.00021	0.00223 0.00086 -0.99970 The structure was solved using dual methods with only the non-overlapping reflections of component 1. The structure was refined using the hklf 5 routine with all reflections of component 1 (including the overlapping ones), resulting in a BASF value of 0.341(2) (Herbst-Irmer & Sheldrick, 1998). The total number of reflections given (_diffrn_reflns_number) is from before merging of data as given by Twinabs. The Rint value given (_diffrn_reflns_av_R_equivalents) is for all reflections of domain 1 and is based on agreement between observed single and composite intensities and those calculated from refined unique intensities and twin fractions (TWINABS (Sheldrick, 2012)).

Table 32. Atomic coordinates and *U_eq_* [Å^2^] for 6

| **Atom** | ***x*** | ***y*** | ***z*** | ***U*_eq_** |
| --- | --- | --- | --- | --- |
| O7 | 0.47053(11) | 0.6155(2) | 0.84967(11) | 0.0312(3) |
| O9 | 0.50162(12) | 0.5897(2) | 0.65745(13) | 0.0314(3) |
| N1 | 0.20334(11) | 0.3006(2) | 0.42011(9) | 0.01741(19) |
| N2 | 0.29090(11) | 0.12448(19) | 0.45321(10) | 0.01657(19) |
| N3 | 0.35271(11) | 0.1295(2) | 0.56383(10) | 0.01707(19) |
| N4 | 0.30323(10) | 0.32722(19) | 0.60876(9) | 0.01503(18) |
| N5 | 0.33251(11) | 0.3935(2) | 0.72983(9) | 0.0170(2) |
| N6 | 0.43969(11) | 0.5400(2) | 0.74280(11) | 0.0197(2) |
| N8 | 0.14621(12) | 0.6292(2) | 0.53975(10) | 0.0198(2) |
| H8A | 0.090(2) | 0.687(4) | 0.479(2) | 0.024 |
| H8B | 0.155(2) | 0.694(4) | 0.610(2) | 0.024 |
| C1 | 0.21176(11) | 0.4311(2) | 0.52201(10) | 0.0150(2) |
| C2 | 0.31875(14) | −0.0571(2) | 0.36598(13) | 0.0219(2) |
| H2A | 0.234481 | −0.110854 | 0.318618 | 0.033 |
| H2B | 0.363972 | −0.192486 | 0.410118 | 0.033 |
| H2C | 0.376490 | 0.009985 | 0.310615 | 0.033 |

*U*_eq_ is defined as 1/3 of the trace of the orthogonalized *U_ij_* tensor.

Table 33. Anisotropic displacement parameters [Å^2^] for 6. The anisotropic displacement factor exponent takes the form: −2π^2^[ *h*^2^*(a*)*^2^*U*_11_ *+ k*^2^*(b*)*^2^*U*_22_ + … + *2hka*b*U*_12_ ]

| **Atom** | ***U*_11_** | ***U*_22_** | ***U*_33_** | ***U*_23_** | ***U*_13_** | ***U*_12_** |
| --- | --- | --- | --- | --- | --- | --- |
| O7 | 0.0288(5) | 0.0322(5) | 0.0288(5) | −0.0163(5) | −0.0092(4) | 0.0021(4) |
| O9 | 0.0286(5) | 0.0280(5) | 0.0396(6) | −0.0025(5) | 0.0110(5) | −0.0116(4) |
| N1 | 0.0208(4) | 0.0180(4) | 0.0129(4) | −0.0008(4) | 0.0006(3) | −0.0003(4) |
| N2 | 0.0188(4) | 0.0162(4) | 0.0146(4) | −0.0018(4) | 0.0020(3) | −0.0023(3) |
| N3 | 0.0196(4) | 0.0151(4) | 0.0158(4) | −0.0010(4) | 0.0003(3) | −0.0006(3) |
| N4 | 0.0182(4) | 0.0143(4) | 0.0116(4) | −0.0004(3) | −0.0012(3) | −0.0005(3) |
| N5 | 0.0200(4) | 0.0187(4) | 0.0113(4) | −0.0005(4) | −0.0013(3) | −0.0033(4) |
| N6 | 0.0180(4) | 0.0159(4) | 0.0237(5) | −0.0042(4) | −0.0026(4) | 0.0007(4) |
| N8 | 0.0251(5) | 0.0172(4) | 0.0153(4) | 0.0000(4) | −0.0030(4) | 0.0044(4) |
| C1 | 0.0161(4) | 0.0159(4) | 0.0123(4) | 0.0008(4) | −0.0004(3) | −0.0018(4) |
| C2 | 0.0249(5) | 0.0200(5) | 0.0216(5) | −0.0087(5) | 0.0056(4) | −0.0025(4) |

Table 34. Bond lengths and angles for 6

| **Atom–Atom** | **Length [Å]** |
| --- | --- |
| O7–N6 | 1.2521(16) |
| O9–N6 | 1.2374(17) |
| N1–C1 | 1.3370(15) |
| N1–N2 | 1.3418(15) |
| N2–N3 | 1.2910(15) |
| N2–C2 | 1.4591(17) |
| N3–N4 | 1.3424(15) |
| N4–C1 | 1.3629(15) |
| N4–N5 | 1.3803(14) |
| N5–N6 | 1.3505(16) |
| N8–C1 | 1.3239(16) |
| N8–H8A | 0.87(2) |
| N8–H8B | 0.86(2) |
| C2–H2A | 0.9800 |
| C2–H2B | 0.9800 |
| C2–H2C | 0.9800 |
|  |  |
| **Atom–Atom–Atom** | **Angle [°]** |
| C1–N1–N2 | 102.42(10) |
| N3–N2–N1 | 116.85(10) |
| N3–N2–C2 | 121.94(11) |
| N1–N2–C2 | 121.15(11) |
| N2–N3–N4 | 102.37(10) |
| N3–N4–C1 | 110.13(10) |
| N3–N4–N5 | 122.92(10) |
| C1–N4–N5 | 126.57(10) |
| N6–N5–N4 | 108.86(10) |
| O9–N6–O7 | 124.06(12) |
| O9–N6–N5 | 122.82(12) |
| O7–N6–N5 | 113.11(12) |
| C1–N8–H8A | 118.6(15) |
| C1–N8–H8B | 120.3(15) |
| H8A–N8–H8B | 121(2) |
| N8–C1–N1 | 127.85(11) |
| N8–C1–N4 | 123.90(11) |
| N1–C1–N4 | 108.24(10) |
| N2–C2–H2A | 109.5 |
| N2–C2–H2B | 109.5 |
| H2A–C2–H2B | 109.5 |
| N2–C2–H2C | 109.5 |
| H2A–C2–H2C | 109.5 |
| H2B–C2–H2C | 109.5 |

Table 35. Torsion angles for 6

| **Atom–Atom–Atom–Atom** | **Torsion Angle [°]** |
| --- | --- |
| C1–N1–N2–N3 | 0.16(14) |
| C1–N1–N2–C2 | −177.14(11) |
| N1–N2–N3–N4 | −0.53(14) |
| C2–N2–N3–N4 | 176.74(11) |
| N2–N3–N4–C1 | 0.69(13) |
| N2–N3–N4–N5 | 174.03(10) |
| N3–N4–N5–N6 | 92.33(14) |
| C1–N4–N5–N6 | −95.46(14) |
| N4–N5–N6–O9 | −3.47(17) |
| N4–N5–N6–O7 | 177.74(11) |
| N2–N1–C1–N8 | 179.52(12) |
| N2–N1–C1–N4 | 0.30(13) |
| N3–N4–C1–N8 | −179.91(12) |
| N5–N4–C1–N8 | 7.05(19) |
| N3–N4–C1–N1 | −0.64(13) |
| N5–N4–C1–N1 | −173.68(11) |

Table 36. Hydrogen bonds for 6

| **D–H⋯A [Å]** | **d(D–H) [Å]** | **d(H⋯A) [Å]** | **d(D⋯A) [Å]** | **<(DHA) [°]** |
| --- | --- | --- | --- | --- |
| N8–H8A⋯O7^#1^ | 0.87(2) | 2.06(2) | 2.9231(16) | 169(2) |
| N8–H8B⋯N5^#2^ | 0.86(2) | 2.08(2) | 2.9306(17) | 172(2) |
| C2–H2C⋯O9^#3^ | 0.98 | 2.56 | 3.2227(19) | 124.6 |

Symmetry transformations used to generate equivalent atoms:

#1: -0.5+X, 1.5-Y, -0.5+Z; #2: 0.5-X, 0.5+Y, 1.5-Z; #3: 1-X, 1-Y, 1-Z;
